# Historical genomics reveals the evolutionary mechanisms behind multiple outbreaks of the host-specific coffee wilt pathogen *Fusarium xylarioides*

**DOI:** 10.1186/s12864-021-07700-4

**Published:** 2021-06-04

**Authors:** Lily D. Peck, Reuben W. Nowell, Julie Flood, Matthew J. Ryan, Timothy G. Barraclough

**Affiliations:** 1 Science and Solutions for a Changing Planet Doctoral Training Partnership, Grantham Institute, Imperial College London, South Kensington, London, SW7 2AZ UK; 2 Department of Life Sciences, Imperial College London, Silwood Park Campus, Ascot, Berkshire, SL5 7PY UK; 3 Department of Zoology, University of Oxford, 11a Mansfield Road, Oxford, OX1 3SZ UK; 4 CABI, Bakeham Lane, Egham, Surrey, TW20 9TY UK

**Keywords:** Comparative genomics, Host adaptation, Fungi, Effector, Proteome, *Fusarium oxysporum*

## Abstract

**Background:**

Nearly 50% of crop yields are lost to pests and disease, with plants and pathogens locked in an amplified co-evolutionary process of disease outbreaks. Coffee wilt disease, caused by *Fusarium xylarioides*, decimated coffee production in west and central Africa following its initial outbreak in the 1920s. After successful management, it later re-emerged and by the 2000s comprised two separate epidemics on arabica coffee in Ethiopia and robusta coffee in east and central Africa.

**Results:**

Here, we use genome sequencing of six historical culture collection strains spanning 52 years to identify the evolutionary processes behind these repeated outbreaks. Phylogenomic reconstruction using 13,782 single copy orthologs shows that the robusta population arose from the initial outbreak, whilst the arabica population is a divergent sister clade to the other strains. A screen for putative effector genes involved in pathogenesis shows that the populations have diverged in gene content and sequence mainly by vertical processes within lineages. However, 15 putative effector genes show evidence of horizontal acquisition, with close homology to genes from *F. oxysporum*. Most occupy small regions of homology within wider scaffolds, whereas a cluster of four genes occupy a 20Kb scaffold with strong homology to a region on a mobile pathogenicity chromosome in *F. oxysporum* that houses known effector genes. Lacking a match to the whole mobile chromosome, we nonetheless found close associations with DNA transposons, especially the miniature impala type previously proposed to facilitate horizontal transfer of pathogenicity genes in *F. oxysporum*. These findings support a working hypothesis that the arabica and robusta populations partly acquired distinct effector genes via transposition-mediated horizontal transfer from *F. oxysporum*, which shares coffee as a host and lives on other plants intercropped with coffee.

**Conclusion:**

Our results show how historical genomics can help reveal mechanisms that allow fungal pathogens to keep pace with our efforts to resist them. Our list of putative effector genes identifies possible future targets for fungal control. In turn, knowledge of horizontal transfer mechanisms and putative donor taxa might help to design future intercropping strategies that minimize the risk of transfer of effector genes between closely-related *Fusarium* taxa.

**Supplementary Information:**

The online version contains supplementary material available at (10.1186/s12864-021-07700-4).

## Background

Fungal diseases have devastated major crop yields throughout history and continue to do so [1, 2]. Large-scale planting of crops generates strong selection for new pathogens to emerge, which leads to further rounds of plant breeding to develop new resistant genotypes. This leads to “boom and bust cycles” that intensify the natural co-evolutionary dynamics of hosts and pathogens. A key goal for sustainable agriculture is therefore to predict disease outbreaks and design robust evolutionary solutions for long-term protection [3]. A first step towards this goal is to understand the genetic and evolutionary mechanisms by which pathogens overcome resistance and infect new host species. Plants have innate defence responses to detect and overcome pathogen attack [4]. In response, an emerging pathogen can evolve new mechanisms to suppress and overwhelm basal plant defences. These could arise by mutation (including gene duplication or loss), recombination and selection operating within a single population, or from hybridization and/or horizontal gene transfer between species to generate new pathogenicity variants [5, 6]. Strong selection to evade plant immunity also leads to host-specificity, whereby pathogens evolve to target particular species or varieties [6]. For example, *Fusarium oxysporum*’s well-studied host-specific *formae speciales* (f. sp.) cause disease on over 120 plant species, including Panama disease of bananas, *F. oxysporum* f. sp. *cubense* [7].

Comparative genomics is revealing the mechanisms that promote rapid evolution of effector proteins and host specificity in fungal pathogens. Effector genes are often found in highly mutable parts of the genome [8]. For example, in ascomycete fungi effectors often occupy AT-rich compartments of the genome with high mutation rates or cluster with transposable elements (TEs), which increase variation via duplications, deletions, insertions and inversions [9–13]. In addition, many ascomycetes have mechanisms to facilitate horizontal transfer of effector genes between taxa either by “pathogenicity islands” in which pathogenicity genes and TEs cluster in chromsosomal segments depleted in GC [14] or by whole mobile chromosomes carrying suites of effectors [9]. For instance, the host-specific virulence protein ToxA was transferred among three wheat pathogens on an 14kb DNA fragment that is rich in transposons and still actively mobile in one of the species [15], whereas the ability of *F. oxysporum* f. sp. *lycopersici* to infect tomatoes derives from a lineage-specific mobile chromosome, which can be transferred experimentally between strains. Pathogenicity can therefore evolve by mutation, recombination and selection operating within a single lineage, or from horizontal gene transfer between strains to generate new pathogenicity variants.

Although comparative genomics has uncovered mechanisms behind host specialisation in several fungal pathogens, exactly how these processes play out during disease cycles remains less clear. Studies have mostly compared contemporary lineages with different host specialisations, rather than tracking genetic changes over time. For example, understanding the roles of within-lineage evolution versus horizontal transfer in generating new effector gene combinations would benefit from comparing genomes before and after boom-bust cycles, as well as between differentially adapted host-specialists.

**Fig. 1:**
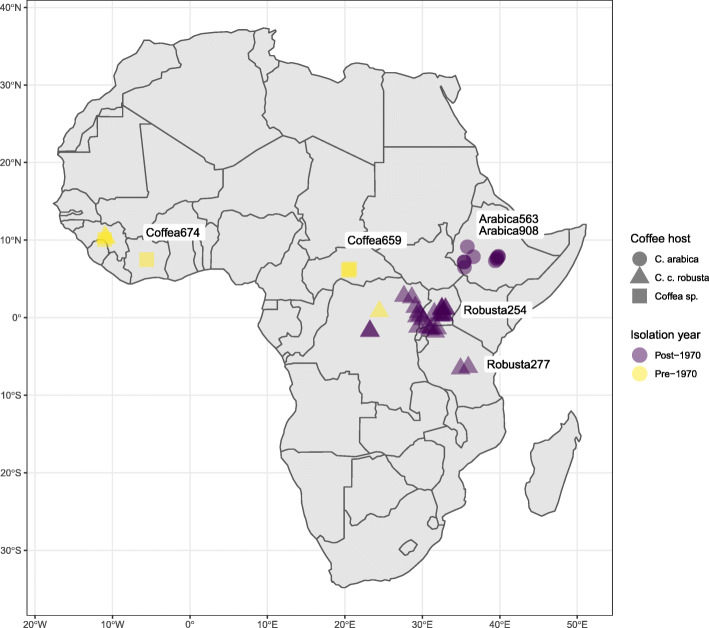
The emergence and spread of *F. xylarioides*. A map of Africa detailing the year collected, country of origin and coffee plant host for the 62 *F. xylarioides* strains in the CABI-IMI culture collection. These strains illustrate the spread west of CWD from the pre-1970s strains to the post-1970s strains, and the emergence of the host-specific arabica and robusta populations. The six strains sequenced in this study are labelled on the map as: *Coffea674*, from Cote D’Ivoire; *Coffea659* from the Central African Republic; robusta254, from Uganda; robusta277, from Tanzania; arabica563 and arabica908, from Ethiopia. Map created in Rstudio 1.2.1335 using the Standard Features package [18] and drawn in ggplot2 [19]

Here, we take advantage of six historic strains collected over 52 years to investigate successive outbreaks and the origin of host specialisation in *Fusarium xylarioides* Steyaert, a soil-borne fungal pathogen that causes coffee wilt disease (CWD). CWD first emerged as a devastating disease of *Coffea excelsa* and *C. canephora* crops in west and central Africa from the 1920s to 1960s [16, 17] (Fig. 1). Improved crop sanitation and breeding programmes successfully reduced its impact but CWD later re-emerged in the 1970s, spreading extensively throughout the 1990s and 2000s [20, 21]. At around the same time that CWD re-emerged on robusta coffee, it was also reported in Ethiopia on “arabica” coffee (*C. arabica*) [22, 23] and *F. xylarioides* was confirmed as the causal agent [24, 25]. By the 1990s, CWD was causing widespread destruction of arabica coffee in Ethiopia, and robusta coffee in the northeast Democratic Republic of Congo (DRC), Uganda and northern Tanzania [20]. It now comprises two host-specific and geographically separated populations, one on *C. canephora* robusta coffee in Uganda, Tanzania and DRC and the other on *C. arabica* in Ethiopia (Fig. 1). Both populations cause significant losses to the coffee cash crop, on Africa’s two most valuable species [26, 27]. *F. xylarioides* therefore offers a unique study system with repeated epidemics and the emergence of two host-specific populations [20, 28, 29]. Critically, historical living strains from the earlier pre-1970s outbreaks as well as the more recent, are optimally cryopreserved in a living state in culture collections.

Previous work described the pathology of the epidemics [20], clarified molecular taxonomy [29, 30], showed reproductive isolation between the host-specialists [28], and reported the first genomes for the robusta population [31, 32]. The genetic basis for successive outbreaks and host-specialisation remains unexplored, however. Wilting occurs when a pathogen proliferates in and blocks the host xylem, so restricting water transport [33, 34]. In order to colonize the xylem vascular system, effector proteins, including carbohydrate-active enzymes such as cellulases and pectinases, are required by the fungus to degrade and penetrate the root system [35, 36]. In *F. oxysporum*, effector proteins behind wilt induction (termed SIX for Secreted In Xylem) are encoded by a single mobile, pathogenicity chromosome [9, 13]. As a result, the same host-specific f. sp.’s can have polyphyletic origins, as the ability to infect a particular host is transferred horizontally [37–40]. Whether similar mechanisms apply for *F. xylarioides* and CWD, and how pathogenicity is restored between successive disease outbreaks, remains unknown. Intriguingly, coffee is intercropped with banana, and *F. xylarioides* and *F. oxysporum* have been co-isolated from roots of both plants in Uganda, and from coffee roots in Ethiopia [29, 41]. Indeed, *F. oxysporum* is able to infect coffee, where it induces a wilt but does not result in the trees’ death [41]. These findings raise the possibility that *F. xylarioides* may have acquired certain pathogenicity genes from *F. oxysporum*, that has facilitated the recent outbreaks on coffee.

To address these questions, we sequenced and compared the genomes of six representative historical *F. xylarioides* strains from the CABI-IMI living culture collection: two strains derive from the 1950s and the pre-1970s outbreak in the Central African Republic and Cote D’Ivoire respectively: *Coffea659* (IMI 127659/ DSMZ 62457) and *Coffea674* (IMI 392674/ CBS 258.52). We call these *Coffea* strains because of their ability to infect multiple *Coffea* species including robusta, and their original hosts are unknown. *Coffea674* is the ex-type and in common with most pre-1970s strains infects robusta and other *Coffea* species but not arabica [28]. *Coffea659* is one of the few strains able to also infect arabica in trials and therefore is a true host generalist [20, 42]. Our remaining four sequenced strains comprise these host-specific populations: two arabica strains (arabica563, IMI 389563 and arabica908, IMI 375908); and two robusta strains (robusta254, IMI 392254 and robusta277, IMI 392277), all collected five years apart between 1995-2005 (Fig. 1). Current evidence from molecular markers and crossing experiments supports the distinctiveness of the arabica and robusta populations [28, 30], but varies with respect to relationships between them and with the initial outbreak’s *Coffea* strains. Differing studies show the 1990-2000s arabica and robusta populations as sister clades [43], or the 1990-2000s robusta population grouped with the *Coffea* strains from the pre-1970s outbreak [29], suggesting it arose from a subset of older strains from the initial outbreak whereas the arabica population is more divergent. Thus, we first tested the hypothesis that the robusta population derived from the pre-1970s outbreak with the arabica population emerging separately. We then compared putative effector genes between the strains. Specifically, we ask (i) whether the 1990-2000s robusta epidemic is genetically different to the earlier outbreak; (ii) whether the host-specialist robusta and arabica populations share similar sets of derived effector genes or whether their similar pathologies evolved independently; (iii) we test whether changes in pathogenicity and host-specialism involved horizontal transfer of effector genes, or was restricted to within-lineage evolution in ancestral sets of effector genes using comparisons with potentially co-occurring and closely related *Fusarium* species, and (iv) explore the possible role of mobile chromosomes or transposable elements in any putative cases of horizontal transfer.

## Results

### General features of the genomes in comparison with other *Fusarium* species

We reconstructed genome assemblies from MiSeq Illumina reads using MEGAHIT [44] ranging in size from 58 Mb in the *Coffea* strains to 61 Mb for the robusta strains and 63 Mb for the arabica strains. The robusta genomes are larger than those previously sequenced (55 Mb), however they have a comparable size if scaffolds <500 bp are excluded (comprising 4Mb in total, Additional file 2: Table S1). Rather than remove these, we included them in our analyses. We assume these genomes to be complete, based on synteny with the previously published *F. xylarioides* genomes (Additional file 2: Figure S1), high levels of contiguity (N50 >40 kb) and 100% proteome completeness based on the presence of BUSCO genes (Table 1).

**Table 1:** Genome statistics for sequenced *F. xylarioides* strains

Name	*Coffea674*	*Coffea659*	Robusta277	Robusta254	Arabica563	Arabica908
Strain number	IMI392674 CBS258.52	IMI127659i DSMZ62457	IMI392277	IMI392254	IMI389563	IMI375908i
Year	1951	1955	2003	1997	2002	1997
Origin	Cote d’Ivoire	Central African Republic	Tanzania	Uganda	Ethiopia	Ethiopia
Host	*Coffea*	*C. excelsa*	*C. c.* robusta	*C. c.* robusta	*C. arabica*	*C. arabica*
Genome Size Mb	57.2	59.6	61.3	60.3	63.4	62.6
No. scaffolds	3969	4843	5484	5369	7078	7264
N50 bp	62845	55667	54978	56291	42705	41762
BUSCO %	100	100	100	100	100	100
GC %	43.3	43.5	43.1	43.1	42.1	42.2
Coding Genes	14529	14732	14852	14588	14589	14654
Coverage %	36	35	34	34	33	33

“Coverage” refers to the proportion of the genome covered by coding regions

**Fig. 2:**
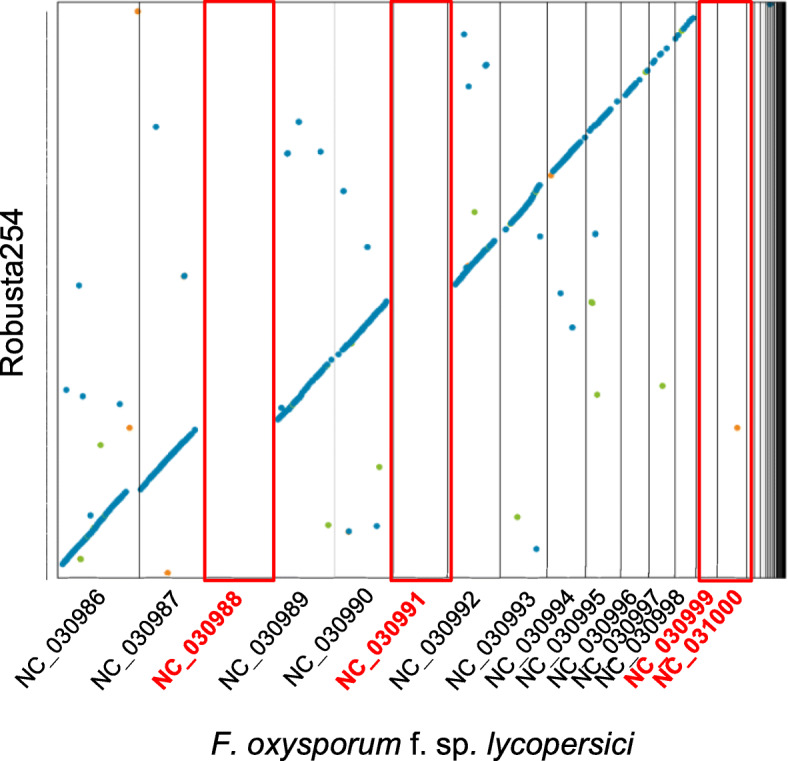
Representative whole-genome alignments of *F. xylarioides* and *F. oxysporum f. sp. lycopersici*. Representative whole-genome alignments of *F. xylarioides* strain robusta277 against the 15 *F. oxysporum f. sp. lycopersici* chromosomes, including the 4 mobile chromosomes, annotated in red, and the 11 core chromosomes shared with sister *Fusarium* species [9]. Each dot represents chromosomal correspondence, with absences representing the absent *Fol* chromosomes. Alignments for each *F. xylarioides* genome are in Additional file 2: Figure S2. Genomes were aligned using Mummer 4.0.0 (http://mummer.sourceforge.net/) with outputs processed using Dotprep.py before visualizing using Dot in DNA Nexus (https://dnanexus.github.io/dot/). Blue indicates forward alignments, green indicates reverse alignments, orange indicates repetitive alignments

**Fig. 3:**
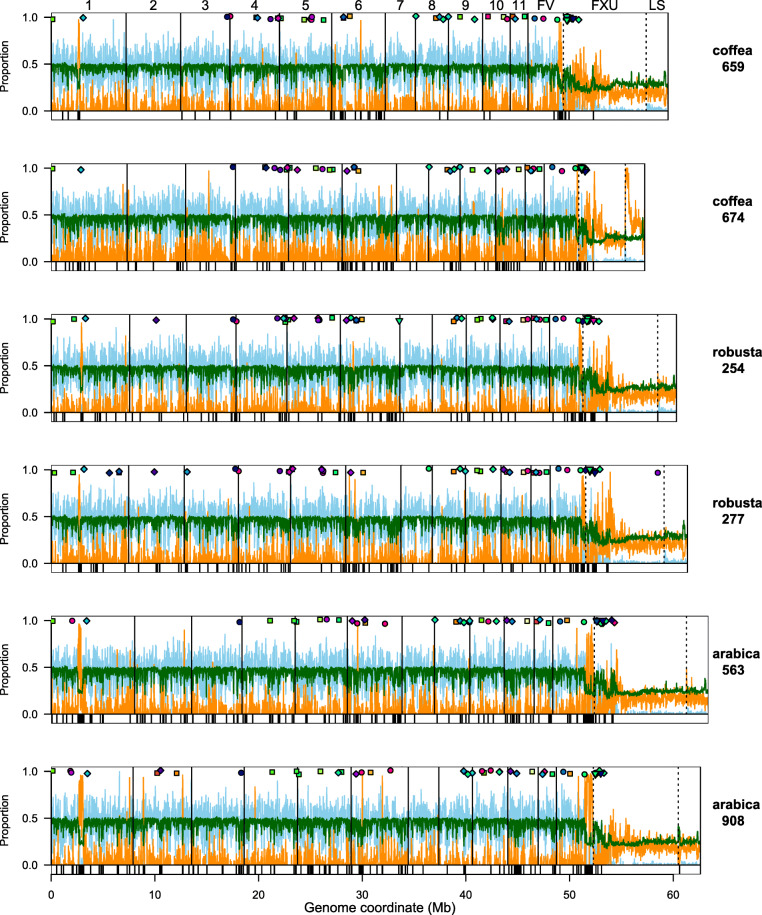
Global view of genome metrics plotted across 20 Kb windows of each *F. xylarioides* genome. Global view of genome metrics plotted across 20 Kb windows of each *F. xylarioides* genome. Light blue = fraction of nucleotides annotated as a gene. Orange = fraction of nucleotides annotated as transposable element. Dark green = %GC. Vertical solid lines demark the 11 inferred chromosomes matching to the *F. verticillioides* genome. Dashed lines demark: FV, scaffolds that are shared with *F. verticillioides* but not assembled into chromosomes; FXU, scaffolds that are absent from *F. verticillioides* but are shared with *Coffea659* and *F. udum*; LS, scaffolds that are not shared with *F. verticillioides, Coffea659* nor *F. udum* and are therefore lineage specific. Coloured points indicate the location of putative effector genes: square = pre-defined in the literature, circle = CAZyme, diamond = small cysteine-rich proteins. Colours allocated across spectrum arbitrarily but same colour indicates belongs to same ortholog. The lower strip on each plot shows Large RIP Affected Regions in black

To evaluate our genomes further, we compared them to published genomes from a range of *Fusarium* taxa (Figs. 2, Additional file 2: Figures S2 and S3, Table S3). *F. xylarioides* has a larger genome than its closely related species from the *Fusarium fujikuroi* Complex (FFC) African clade (also known as the *Gibberella fujikuroi* complex GFC) [45], *F. udum* (56.4 Mb, [46]), which causes wilt on pigeon pea, and that of *F. verticillioides* (41.7 Mb, [9]), which is a non-wilt plant pathogen of maize. The genomes are similar in size, however, to the more distantly related *F. oxysporum* f. sp. *lycopersici (Fol)* strain 4287 (60 Mb, [9]). Representative whole-genome alignments revealed the presence of the 11 syntenic core chromosomes shared by *F. verticillioides, F. oxysporum* and more distantly-related *Fusarium* taxa [9] in *F. xylarioides* (Additional file 2: Figures S2 and S3), and the additional genomic material compared with *F. verticillioides* (Additional file 2: Figure S3). To understand precisely which *F. xylarioides* scaffolds matched these syntenic chromosomes, we used reference-guided scaffolding to orient our contigs into chromosomes based on the de novo long-read sequencing *F. verticillioides* assembly [9]. This resulted in 85% of our contigs for each genome mapped to its syntenic chromosomes and un-aligned scaffolds (labelled “FV”) of *F. verticillioides*, with the remaining 15% comprising shorter un-aligned scaffolds (Fig. 3). We then classified these un-aligned scaffolds based on their presence and absence across other FFC species: those which are absent from *F. verticillioides* but which are present in *F. udum* and the historic *Coffea659* strain are labelled “FXU” (*F. xylarioides* and -*udum* specific); those which are absent from *F. verticillioides* and *F. udum* and are shared with *Coffea659* are labelled “FXS” (*F. xylarioides*-specific); and those which are not shared with *Coffea659* and are unique to each *F. xylarioides* strain are labelled “LS” (lineage-specific). The FXS scaffolds make up <0.5% of the genomes, while LS scaffolds are 3.5% and FXU scaffolds are 12% (Table 1). Genome size differences in the arabica and robusta strains are largely explained by the FXU and LS scaffolds (11 Mb and 9 Mb respectively) (Additional file 2: Table S2).

*F. xylarioides* and *F. udum* are the only known members of the FFC to infect their target hosts with a wilt disease [47, 48]. To test whether *F. xylarioides* might have evolved new wilt capabilities by acquiring a pathogenic chromosome from *Fol*, we mapped the *F. xylarioides* genomes to the *Fol* genome assembly. Chromosomes 3, 6, 14 and 15 in the *Fol* genome are supernumerary, or mobile, as well as parts of chromosomes 1 and 2 [9]. Chromosome 14 is also pathogenic, housing all known *Fol* wilt effector genes. We found no large-scale matches to any of the fully mobile chromosomes in *F. xylarioides* (Fig. 2 and Additional file 2: Figure S2), ruling out the recent transfer of the whole *Fol* mobile pathogenic chromosome. However, there was a significant excess of smaller matches between the LS and FXU scaffolds and the *Fol* mobile chromosomes (Additional file 2: Figure S4, Pearson’s chi-sq test: p <0.001). Potentially, this is consistent with smaller pieces having been transferred: 7% of the FXU scaffolds and 20% of the LS scaffolds matched to the four fully mobile *Fol* chromosomes (3, 6, 14 and 15), whereas <2% matched to non-mobile chromosomes. With short-read sequencing we cannot infer the chromosomal assembly of the LS and FXU scaffolds nor rule out that *F. xylarioides* has novel mobile chromosomes not closely related to those in *F. oxysporum*. However, the pattern of strong hits to parts of the *Fol* mobile pathogenic chromosome but lack of hits to the majority of it does raise the possibility of transfer of smaller genome regions that we explore further below using alternative evidence.

Annotation of genes and transposable elements (TEs) in the MEGAHIT assemblies reveal the whole genomes, along with *F. udum*, contain on average 14,650 predicted protein-coding genes, covering 34% of the genomes and 5 Mb of repeats, covering 9% (Table 1 and Additional file 2: Table S1). Within our reference-guided assemblies, we found the FXU and LS scaffolds differ considerably in overall genomic content. The 11 core chromosomes along with the FV scaffolds are gene-rich and repeat-poor, whereas the FXU and LS scaffolds are gene-poor and repeat-rich (Fig. 3). The distribution of genes attributed to particular functional classes was highly concordant among the six *F. xylarioides* strains, and within each core chromosome except chromosome 11, which is enriched for putative functions related to stress response and protection from reactive oxygen species (Additional file 2: Figure S5). Interestingly, genes on FXU and LS scaffolds are also enriched for the putative function ‘phage major capsid proteins’ (Additional file 2: Figure S5b), which could be involved with movements of mobile elements. In terms of TEs, we found consistent content across both the MEGAHIT and our reference-guided genome assemblies of 5 Mb of interspersed repeats made up of simple repeats, retroelements and DNA transposons. We found slightly less (4 Mb) if we analysed the raw reads which shows that our methods find more TEs than an assembly-free method (Additional file 2: Table S1). In contrast, the FXU and LS scaffolds are enriched for transposable elements, which overall comprise 24% of DNA in these scaffold groups, compared to 5% in the 11 core chromosomes. Within this, retrotransposons have contributed to genome expansion in *F. xylarioides*, as is commonly observed in other fungi [13], making up 16% of DNA in these scaffold groups, compared to 3% in the 11 core chromosomes. The LS scaffolds are further enriched for DNA transposons, with 150% more than is found in the FXU scaffolds and 360% more than in the core chromosomes. The proportion of the whole genome made up of transposable elements also varies among species. Some of these differences could reflect bias due to different assembly methods. For instance, *F. oxysporum* has a high repetitive sequence and TE content: we calculated 6.2 Mb TEs from the *Fol* long-read assembly (Additional file 2: Table S1), which is considerably more than the number we calculated from the *Fol* raw reads (1.2 Mb, Additional file 2: Table S1). It is possible that future long-read sequencing would also reveal more TEs in our *F. xylarioides* strains. Despite this potential bias, however, *F. xylarioides*, has nearly 1000% more TEs than recovered from the whole chromosome assembly of *F. verticillioides* (Additional file 2: Table S1).

The *F. xylarioides* genomes display another common structural feature of plant pathogenic ascomycetes, namely the presence of AT-rich genome blocks. Specifically, the Repeat-Induced Point-like (RIP) mutations pathway is a genome defence mechanism against the invasion of mobile transposons that has been linked to enhanced mutagenesis of effector genes in some plant pathogens [8]. It is unique to ascomycete fungi and mutates cytosine bases to thymine in repeated genome sequences [49]. This leads to AT-rich sequences that are typically low in coding gene content, and can segment the genome into equilibrated compartments (as in *Leptosphaeria maculans*, [11]). Across both MEGAHIT and reference-guided assemblies, we find significant evidence for AT-rich blocks (averaging 21.5% GC), which are spread throughout the core chromosomes and FV scaffolds, and the FXU and LS scaffolds (Fig. 3). On average, these blocks are significantly poor in genes but rich in repeats, in common with patterns in other fungi. We find that RIP regions make up >33.5% to >40% of each genome, with >7.7% to >8.5% of the genomes in so-called Large RIP Affected Regions (LRARs, (Fig. 3). Having described the broad features of our genomes we now address our questions concerning the multiple outbreaks.

### The arabica population arose independently from the robusta and *Coffea* strains

**Fig. 4:**
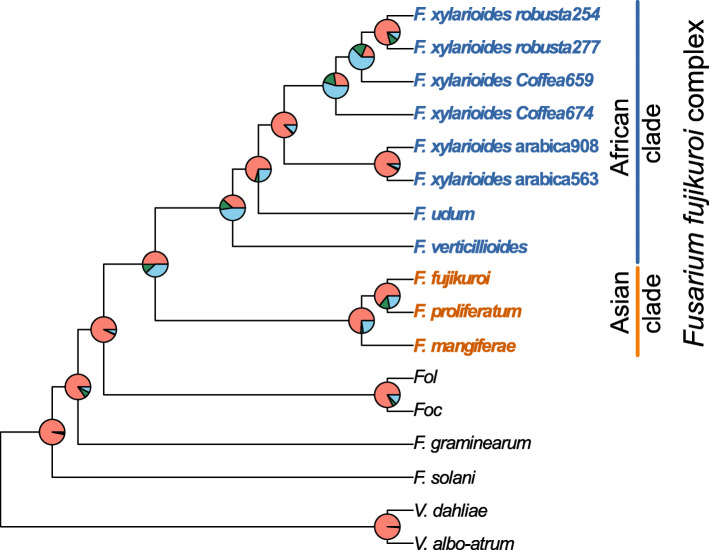
Phylogenetic relationships between *Fusarium* species. Phylogenetic relationships between *Fusarium* species reconstructed from 13 782 orthogroups support monophyly of the *F. xylarioides* clade, with little consistent support for alternate topologies. Strain accession numbers are in Additional file 2: Table S3. The brackets denote the FFC, with the Asian (blue) and African (red) clades. Pie chart colours: pink = proportion of genes (orthogroups) recovering the depicted node; dark green = the proportion of genes recovering the second most common topology; light blue = the proportion of genes recovering all other topologies

Our genome data supports the previous hypothesis that the arabica and robusta populations emerged independently within *F. xylarioides* [29]. Gene annotations from the *F. xylarioides* and *F. udum* strains together with ten other published *Fusarium* and *Verticillium* wilt genomes were used to identify 18 569 orthologous gene sets, encompassing 25,0056 genes or 95.6% of all annotated genes. The species tree based on the concordance of 13,782 gene trees of all ortholog groups supports the established order of the FFC [45] as well as the monophyly of *F. xylarioides*, with over 87% of genes supporting monophyly of the clade (Fig. 4). No alternate topology was consistently found for the remaining gene trees (the next most common was supported by just 1.1% of genes), confirming that *F. xylarioides* did not originate by hybridisation or major influx of genes from other taxa. Within the *F. xylarioides* clade, the arabica strains are recovered as a sister clade to the robusta and *Coffea* strains with high concordance of gene trees, consistent with them constituting genetically isolated taxa. While the two robusta strains are also monophyletic with high concordance, there is little concordance for their branching order with respect to *Coffea* strains: loci vary in whether robusta strains are closer to *Coffea659* or to *Coffea674*. Whilst analysis of more strains is needed to confirm this conclusion, it fits the hypothesis that the robusta population emerged from within a wider recombining population of the more genetically diverse *Coffea* isolates, whereas the arabica population is a more divergent lineage within the *F. xylarioides* clade.

**Fig. 5:**
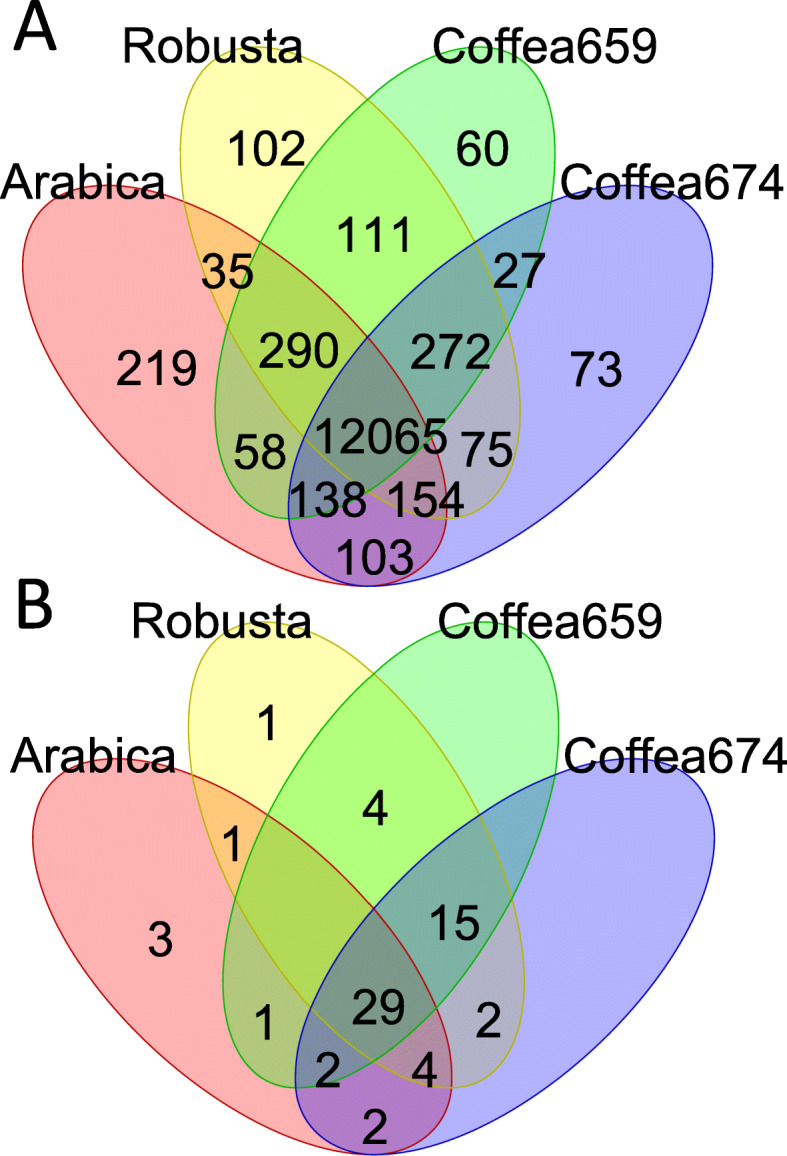
Gene sharing across the *F. xylarioides* strains. a) Orthogroups shared between *F. xylarioides* arabica, robusta and the two *Coffea* strains. Drawn using 13 782 orthogroups (excluding 449 *F. xylarioides* robusta orthogroups and 175 *F. xylarioides* arabica orthogroups that differed in presence/absence between the two strains of each population. The two *Coffea* strains are more divergent (with 1123 orthogroups that differ in presence/absence between the two strains) and so are drawn separately. The two arabica strains (563 and 908) share 13 062 genes and the two robusta strains (254 and 277) share 13 104 genes in total. b) Putative effectors shared between *F. xylarioides* arabica, robusta and the two *Coffea* strains. Drawn using 64 putative effector proteins that differed in presence/ absence across the host-specific populations

This conclusion is further supported by patterns of presence and absence of genes. Over 96% of single gene copies were shared between the two arabica and between the two robusta strains respectively, while there is less similarity between other *F. xylarioides* comparisons: *Coffea*-robusta, 93%; *Coffea-Coffea*, 92%; *Coffea*-arabica, 92%; arabica-robusta, 91%; and *F. xylarioides*-*F. udum*: 84% (Fig. 5a). The robusta strains share a significantly higher proportion of orthologous gene sets with *Coffea659* (SuperExactTest, p <0.001), and they generally display more concordance with the *Coffea* strains, while the arabica strains have the most unique orthogroups. The arabica strains share slightly more genes with the ex-type strain *Coffea674* than with *Coffea659* despite the latter being the only known strain able to infect both arabica and robusta coffee [29, 50].

### A set of putative effector proteins for *F. xylarioides*

The proteome of plant pathogens includes pathogenicity factors and effector proteins, which are secreted to enable pathogen entry, survival and effective colonisation in their host. Thus fungal effector proteins are typically under positive selection and do not show conserved protein sequences [8, 51]. Therefore, we adopted a multi-dimensional approach to look for putative effector proteins, namely those which enable the pathogen to become established in its host and trigger the onset of symptoms and a host defence response. We describe three classes: *(i)* those known from other fungal pathogens, *(ii)* small and cysteine-rich proteins, *(iii)* carbohydrate-active enzymes, and *(iv)* genes with class II TEs in their promoter regions, specifically including the presence of *miniature impala (mimp)* transposons which are associated with *F. oxypsorum* effector genes [13]. We also annotated the following characteristics: presence of positive selection indicating genes which are under accelerated evolution; presence of a signal peptide for secretion, because it is assumed that effector proteins need to be secreted during host colonisation; genes which are present in an AT-rich genome block as an indicator of potential transposon invasion; the predicted function based on the Pfam domain; and genes which are absent from sister FFC species and share *F. oxysporum* as the closest match with a percent identity above 90%. Altogether, this process identified 64 putative effector genes, which were distributed across scaffolds and the reference-guided scaffold assemblies, and were mostly recovered in the same location in each strain (Fig. 3). Because effector genes have not been investigated previously in *F. xylarioides*, we first describe general findings for each category of effector genes, before focusing on the differences between the different host-specific populations.

#### Putative effector genes known from other species

**Fig. 6:**
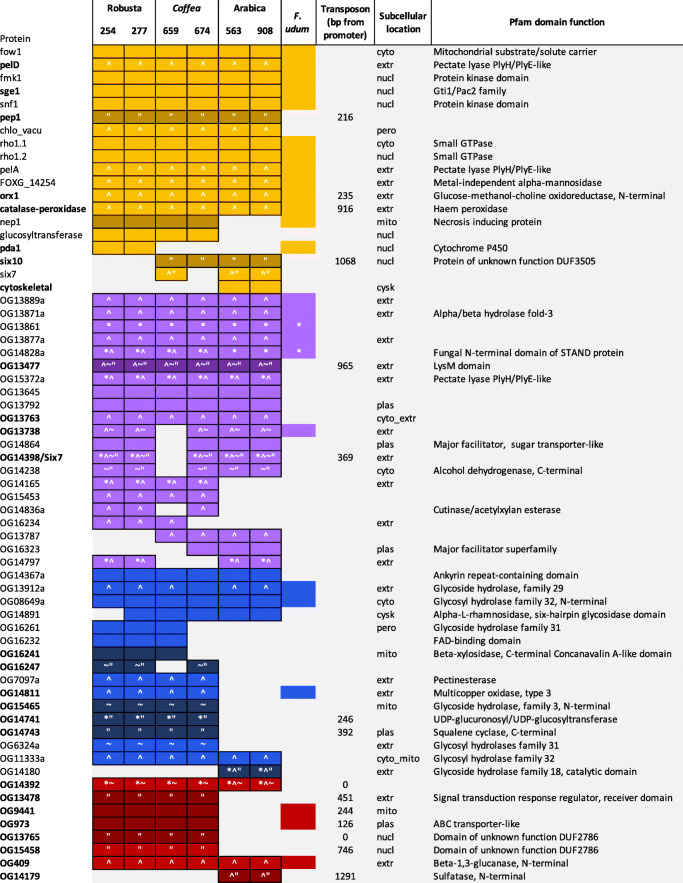
Putative effectors’ characteristics and presence or absence across *F. xylarioides* strains and *F. udum*. The four effector classes are shown in: yellow for predefined effectors; purple for small and cysteine-rich effectors; blue for carbohydrate-active enzymes; and red for transposon-adjacent effectors. The presence of transposons is represented by names in bold with its distance from the genes promoter described if less than 1500bp (if not, the transposon is over 1500bp away), genes under positive selection by an asterisk, secreted proteins with a signal peptide by a caret, genes in an AT-rich block by a tilde, genes with evidence of horizontal transfer from *F. oxysporum* are a darker shade and function is represented by the Pfam domain, where a hit was returned. Genes which are absent from the FFC with *F. oxysporum* the closest match with a percent identity (%) >=90 are represented by a quotation mark

We found BLAST matches in *F. xylarioides* to 19 known effector proteins previously characterised in *F. oxysporum* and other wilt-inducing pathogenic fungi, including some that are only recently discovered and unnamed (Additional file 2: Table S4, Fig. 6). The effector genes *fow1, fmk1, snf1, pelD, chlo-vacu, rho1* (here with two copies *rho1.1* and *rho1.2*), *pep1, pelA, orx1, FOXG_14254* and *catalase-peroxidase* as well as the transcription factor Sge1 were found in all six *F. xylarioides* strains, with *nep1, glucosyltransferase, pda1, six7, six10* and *cytoskeletal* found across the host-specific pairs. The majority of the well-characterised SIX effectors from *Fol* chromosome 14 are absent in both *F. udum* and all *F. xylarioides* strains, with the exceptions of the recently described Six10 and Six7 proteins [13] which are present in arabica and *Coffea* strains, and the transcription factor Sge1, which is required for the expression of SIX genes [7], is found in all strains.

#### Small and cysteine-rich secreted proteins

Many fungal effector proteins are small (<400 amino acids), cysteine-rich (>4 cysteine residues) and secreted [8, 52]. Searching our annotated proteomes for these properties discovered over 2,500 small, cysteine-rich proteins in each strain, of which over 500 also have a signal peptide and a signal peptide cleavage site. Focusing on single copy genes for ease of comparison left 132 putative effectors in 21 orthologous groups shared across *Fusarium* and *Verticillium* (Fig. 6). Patterns of presence and absence were variable among these genes: ten were recovered in all *F. xylarioides* strains. Many of these genes had no recognisable Pfam domain function identified by InterPro (Fig. 6). This is expected because there are large numbers of unknown small and cysteine-rich proteins secreted in the apoplast (as part of the water-transport pathway) which are typically species- or strain-specific with no known function [8]. However, the orthogroup OG13477, found in all *F. xylarioides* strains, matches the LysM-containing chitin oligosaccharide protein, which in *Cladosporum fulvum* is required for scavenging of chitin oligosaccharides as a conserved fungal defence to enable undetected invasion [53]. Orthogroup OG14398 displayed a match, albeit with a relatively low BLAST score, to a number of *six7F. oxysporum* genes and protein sequences (e.g. MG647014.1) (notably not the *Fol* 4287 sequenced genome used for this comparative analysis), which might indicate that it is a divergent type of Six7 effector compared to previously described copies. Other functional annotations reveal a recurring theme of pectin degradation (expanded in next section): OG13871 is a hydrolase; OG15372 is a pectin lyase and OG14836 a carbohydrate esterase 5 with a carbohydrate binding module (CBM1); while OG14864 and OG16323 are major facilitator proteins involved in the transport of solutes, which could influence wilting.

#### Carbohydrate-active enzymes

Carbohydrates such as cellulose and pectin in plant cell walls provide the main source of carbon for fungal pathogens [54] and so carbohydrate-active enzymes (CAZymes) are thought to be important in the infection pathway. We therefore searched for CAZymes restricted to some or all of the wilt-inducing species of *Fusarium* and *Verticillium* and classified their carbohydrate-binding modules (CBMs) to characterise specificity for particular polymers. Our search found 56 genes of interest belonging to 16 orthogroups (Fig. 6). As a whole, *Fusarium* is broadly enriched for lytic enzymes involved in carbohydrate metabolism along with other ascomycete fungi [55, 56] (Additional file 2: Table S5), while the vascular wilt inducers show enrichment in certain gene families (Additional file 2: Figure S10), also reported by [10]). Fungal growth on pectin requires either the combined activities of pectin esterases and polygalacturonases or the sole activity of pectate lyases [34, 57], and all three enzymes are known from other wilt-inducing fungi [58–60]. Interestingly, each share orthologous groups across the wilt-inducing strains analysed in this study (Additional file 2: Figure S10). A polygalacturonase enzyme (glycoside hydrolase (GH) family 28) from *F. oxysporum*, has expanded by one copy number in all *F. xylarioides* strains. Pectate lyase carbohydrate-binding modules CBM66 and CBM38, which bind a fructose-hydrolysing GH32, and chitin-cleaving CBM18 and CBM50 are also found across all six *F. xylarioides* strains (as well as *F. udum* and *Fol*). In contrast pectin esterases have expanded differentially among different *F. xylarioides* strains (see next section).

#### Putative effectors with class II TEs in their promoter regions

Effector genes in *F. oxysporum* are associated with Class II TEs [13]. There is a certain type of class II TE known as a *mimp* for *miniature impala* (part of the miniature inverted-repeat transposable element (MITE) family) which was first described in *F. oxysporum* [61]. In *Fol*, [13] reported that *mimps* (along with several other newly described types of class II TEs) are mostly found on the accessory chromosomes and within the promoter regions of the SIX effector genes [13]. Furthermore, *mimps* have recently been found in a few FFC species [62] and phylogenetic evidence supported repeated transfers between *F. oxysporum* and FFC species. Consequently, we looked for a final class of putative effectors that met four criteria: they were present in both strains for each host-specific population; they contained *mimps* or the newly described transposon families from [13] in their promoter regions (defined as 1500 bp upstream from the start codon); they were on FXU or LS scaffolds; and they matched regions of *Fol*’s mobile chromosomes. This resulted in eight additional putative effectors to supplement those identified by our three original criteria, with three containing mimps, two containing *hop3*, two containing *yaret2* and one with *yaret1* DNA transposons (Fig. 6). Additionally, 15 putative effectors from the predefined, small and cysteine-rich and CAZyme classes also shared a scaffold with a transposon, with four genes across the robusta and *Coffea* strains (*catalase peroxidase, og14741, og14743, og14398*) and *six10* across the arabicas with *mimps* in their promoter regions. The potential role of transposition on effector gene evolution is investigated further in section 3.6 – for now we treat them simply as another class of putative effector genes.

### Host-specific populations differ in their complement of putative effectors

Effector gene presence and absence recapitulated findings from the whole genome analyses: both strains from the same host-specific population nearly always share the same effector gene complement, and the robusta population and *Coffea* strains share greater overlap in effector gene complement (SuperExactTest, p=0.001) than either do with the arabica population (Fig. 5b). Based on this significant association, we looked for changes that might have led to specialisation on robusta coffee and increased virulence, and found just 1 effector unique to the robusta strains, *pda1*. This gene encodes the enzyme pisatin demethylase, which has been shown to detoxify the plant defensive compound (phytoalexin) pisatin by *F. oxysporum* f. sp. *pisi* in peas, and with different alleles present across different *F. oxysporum* host-specific f. sp.’s [39, 63, 64]. Interestingly, in sugarbeet one *pda1-a* allele (accession AY487143.1) was only found in pathogenic strains [63], although the gene copy found in this study is 97% similar to a *pda1* from *F. oxysporum f. sp. phaseoli* and is only 62% similar to the pathogenic sugarbeet allele. The enzyme could have a direct role against related phytoalexin compounds in coffee or enable growth of robusta strains on alternative hosts. However, given that both *Coffea674* and *Coffea659* lack *pda1* it is unlikely this gene is essential for robusta infection. Indeed, with the exception of *pda1*, the robusta population is more unique in the genes that it lacks than in the genes it has gained when compared with the *Coffea* strains. Because plant immune systems are triggered by detection of secreted effector proteins, the loss of genes might also be important for pathogenicity on different hosts, should plants evolve to recognise certain effectors. Four orthogroups that are present in one or both *Coffea* strains are missing from the robusta strains: *six7, six10, OG13787* and *OG16323*, so absence of these genes could be further explored as a possible cause of enhanced pathogenicity on robusta.

In contrast, the arabica population is divergent to both the *Coffea* strains andtherobusta population in its complement of effector genes. Arabica strains share three unique effectors: Cytoskeletal, the CAZyme OG14180 and OG14179 with a TE in its promoter region. The wilt-specific Cytoskeletal protein was recently described in *Fol, V. dahliae* and *V. albo-atrum* and found to be absent from non-wilt inducing *Fusarium* species [10]. The CAZyme *OG14180* encodes for a GH18 chitinase that has an insertion under positive selection relative to the matching copy in the *Fol* genome, potentially indicating divergent function within the arabica population. In addition, the arabica strains lack 17 effector orthologs found in one or both *Coffea* strains: *gluco, nep1*, five small cysteine-rich proteins including a hydrolase and a pectate lyase, and ten CAZymes including five glycoside hydrolases, another glucosyltransferase *OG14741*, and a CE8 pectinesterase *OG7097*. Interestingly, two of these hydrolases, *OG7097* and *OG6324* have apparently inactivated with 20-30% shorter gene copies which were annotated as two separate genes in both arabica strains: in *OG7097* the arabica strains are missing a 500 bp piece of the gene compared with the robusta strains and a stop codon has truncated it into two genes; and in *OG6324* arabica563 has two *Copia* long terminal repeat (LTR) TE insertions and arabica908 has three unknown TE insertions in the middle of this gene, splitting it and presumably removing functionality. Interestingly, the arabica strains have 20% more copies of these repeats than the robustas, and 35% more than the *Coffea* strains. The two glucosyltransferases (predefined *gluco* and *OG14741*) are found across the robusta and *Coffea* strains, *F. oxysporum* f. sp.’s including *Foc, Fol*, *V. dahliae* and *V. albo-atrum*. The predefined *gluco* glucosyltransferase is required for full pathogenicity in *V. dahliae* [10], and in *F. xylarioides* it shares a scaffold with the small cysteine-rich putative effector *OG14165*, which is also only present in the robusta and *Coffea* strains (see below). The Nep1 protein induces necrosis and ethylene production in host plants [65] with its family expanded in *Fol* and *V. dahliae* and purportedly responsible for their broad host ranges [66]. It is noteworthy that no putative CAZyme effectors are shared exclusively between arabica and the *Coffea* strains, whereas the robusta strains share seven with the *Coffea* strains.

While these differences confirm separation of the arabica population from the other strains, a few putative effectors displayed contrasting affinities. The four genes highlighted as absent in robusta strains are cases of sharing between *Coffea* and arabica strains. Of these, *six7* is shared with *Coffea659*, the only *Coffea* strain that is also able to infect arabica coffee [29, 50], and therefore of possible interest as pathogenicity factors for growth on arabica coffee. Just one gene, *OG14797*, is shared by robusta and arabica populations but absent in both *Coffea* strains. Interestingly, this gene gave a significant signal for positive selection and displays considerable amino acid divergence between the two forms (91% Pairwise Identity). However, both copies share *F. anthophilum*, a member of the American FFC clade [45], as closest relative (with nearly 80% similarity), while a diverged copy in *F. verticillioides* is closer to *F. nygamai* (84% similarity), another African FFC clade member [45]. It is possible therefore that this gene diverged rapidly under positive selection for differential function in each population or that these represent separate acquisitions from alternative sources, despite the same closest BLAST match for *F. xylarioides*. Either way, there is no evidence for substantial recent exchange of effector genes between arabica and the other populations, consistent with genome-wide evidence for their isolation.

We also looked for changes in the number of copies of CAZyme gene families shared by orthologous groups among *F. xylarioides* strains as a possible cause of changes in the ability to degrade plant cell walls and access carbon from their host. Excluding the groups which matched with non-wilt inducing species, we found differences in vascular wilt-inducing plant cell wall-degrading gene families between robusta and arabica populations, with the *Coffea* strains, as before, more similar to robusta. Plant cell walls contain pectins, celluloses, hemicelluloses and other polysaccharides which, along with simple sugars such as mannans, wilt fungi can utilise as a carbon source [34, 67]. Comparing robusta and *Coffea* with arabica, different CAZyme gene families which share functions have expanded across them: the pectinase families CE8, GH78 and GH88 in robusta and *Coffea*, and PL1 in arabica; the xylanase, xylan being a component of hemicellulose, families CE5, GH3, GH43_11, GH43_24 and CBM35 in robusta and *Coffea*, versus CBM42 and GH43_26 in arabica; and the mannanase families CBM13 and GH134 in robusta and *Coffea*, and GT22 in arabica. Whilst not specific to pectin, CBM1-containing carbohydrate esterases that bind cellulose and hydrolyse xylans, mannans and pectins [68–70] were only present in robusta strains and *Coffea674*. While we cannot infer the functional effects of these changes on the infection process, they are suggestive of differences in carbon usage between the two host-specific populations.

Although the main differences among strains are apparent in gene content, we also found cases of divergent selection acting on the amino acid sequence of some shared genes: for example, OG13861 and OG14828 found in all strains, and OG14398 found in all strains except *Coffea659* (Fig. 6). The latter ortholog is the potentially divergent form of *six7* identified as a small cysteine-rich protein and displays amino acid divergence (92% Pairwise Identity) in a pattern consistent with strain host-specificity.

### Several effector genes have been acquired horizontally by transposable elements

Previous work has shown the importance of horizontal gene transfer in the spread of new fungal plant pathogens, and we therefore screened our panel of putative effectors for evidence of acquisition by horizontal transfer. We especially considered the hypothesis that effectors associated with wilt-formation might have been acquired from *F. oxysporum*. We blasted each effector against a panel of *Fusarium* genomes encapsulating the FFC (the closest relatives of *F. xylarioides*), a wide variety of *F. oxysporum* f. sp.’s (as possible sources of horizontally acquired DNA) as well as distantly related *Fusaria*, and retrieved hits with E >-20 and length >90%. We then reconstructed gene trees with alignments of matching genes retrieved from each genome. We expect vertically inherited genes in *F. xylarioides* to group with FFC lineages, as in the core phylogeny (Fig. 4), whereas horizontally acquired genes to group with *F. oxysporum*.

**Fig. 7:**
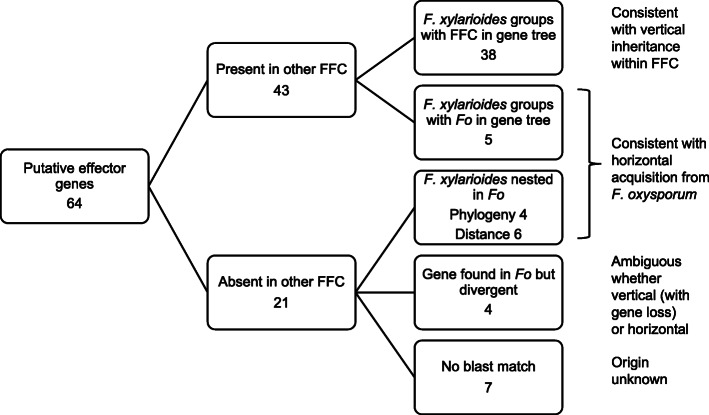
Horizontal gene transfer between *F. xylarioides* and *F. oxysporum*. Decision tree showing the numbers of putative effector genes in *F. xylarioides (Fxyl)* displaying different patterns in their phylogenetic relationships, focusing on possible horizontal acquisition from *F. oxysporum (Foxy)*. FFC = *F. fujikuroi* complex that *F. xylarioides* belongs to. Interpretations consistent with each pattern are shown on the right

**Fig. 8:**
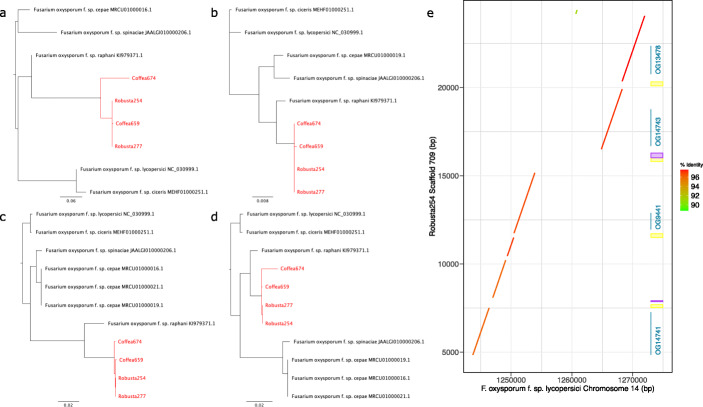
Four putative effector genes on the robusta254 scaffold and their phylogenetic trees. **a** Phylogenetic tree for *og14741*, **b** Phylogenetic tree for *og9441*, **c** Phylogenetic tree for *og14743*, **d** Phylogenetic tree for *og13478*. For each gene, the FFC is absent, *F. xylarioides* is nested within *F. oxysporum* and *F. oxysporum* f. sp. *raphani* is the closest match (also shown in Additional file 2: Figure S7). All branch support values = 100%, all trees drawn in Geneious 9.1. **e** The four effector genes are surrounded by *mimps* and DNA transposons on a robusta254 scaffold which shares a high (>96%) nucleotide sequence identity with the mobile and pathogenic *Fol* chromosome 14. Similar scaffolds with the same four putative effector genes are present in robusta277, *Coffea659* and *Coffea674*. Alignments were made with nucmer (MUMmer3). The blue annotations indicate the effector genes, the yellow annotations indicate *mimps* and the purple annotations indicate DNA transposons

Across 64 effectors (Fig. 7), seven display no BLAST hit and we consider their origin unknown. All others have positive hits in at least one *F. oxysporum*. Most of these (45) also display at least one hit in an FFC genome, and indeed in 40 of these effectors the *F. xylarioides* sequences group with FFC sequences, which is consistent with vertical inheritance. Note that nine of these cases had an FFC match only in *F. udum*, the wilt-forming sister lineage to *F. xylarioides* (Additional file 2: Table S8, cases annotated with *). Potentially these cases could represent horizontal acquisition from *F. oxysporum* in the common ancestor of *F. udum* and *F. xylarioides*, but we focus on putative transfer into the *F. xylarioides* lineage here and so label these as vertical. The remaining 5 effectors, out of 45 with positive hits in the FFC, group more closely to *F. oxysporum* lineages with strong support (approximate Likelihood Ratio Test, aLRT support >0.95, Additional file 2: Table S8), consistent with horizontal transfer. Among the remaining 14 effectors (of the original 64) with no hits detected in FFC genomes, *F. xylarioides* copies nest within the variation observed among *F. oxysporum lineages* in 10 cases (based on phylogenetic and distance criteria, see Methods), which is again consistent with horizontal transfer (e.g. Fig. 8a-d). In the final 4 cases, *F. xylarioides* sequences are highly divergent from all *F. oxysporum* sequences and so a hypothesis of recent horizontal acquisition is not strongly supported. Those genes could have been vertically inherited but secondarily lost in all other FFC lineages, or diverged so much that they returned no match in other genomes, or been acquired horizontally from lineages not included in our sample.

Among the 15 putative effectors with evidence for acquisition by transposition, two are shared by all six *F. xylarioides* strains. Of these, one is the putative divergent form of Six7 (OG14398) that displays evidence of divergent selection between host-specific strains and the other (OG13477) matches a LysM domain. Three effectors are in the arabica strains, with one, another SIX protein (Six10) shared by arabica and the *Coffeas*, and two effectors unique to the arabica population and both involved in hydrolysis. The remaining ten effectors are shared by the robusta and at least one of the *Coffea* strains but not arabica. Six of these have recognised functional annotations:Nep1, involved in necrosis; OG16241 and OG15465, hydrolysis of sugars; OG14741,glucosyltransferase; OG14743, squalene-hopene-cylase; OG13478, involved in signalling; OG973, transporter protein. Thus HGT from *F. oxysporum* appears to have played a greater role to the emergence of the robusta and *Coffea* host-specific strains than in the arabica population. Of the *formae speciales* which consistently match, *F. oxysporum* f. sp. *raphani* shares a percent identity >98% with four of these HGT effectors only found in the robusta and *Coffea* strains (Additional file 2: Figure S7).

We further interrogated the 15 possible cases of horizontal transfer from *F. oxysporum* by investigating the flanking regions in our MEGAHIT assemblies. Of particular interest is one scaffold found in the robusta and *Coffea* genomes, which contains these 4 putative effectors matching *F. oxysporum* f. sp. *raphani* with evidence for horizontal transfer (OGs 9441, 13478, 14741 and 14743) in each strain (Fig. 8a–d). Additionally, this scaffold is absent from *F. verticillioides* and designated as FXU in section 3.1, for each strain. This 20 Kb scaffold displays high (>97%) identity with *F. oxysporum* (Fig. 8e), and further features suggestive of horizontal transfer described in section 3.6. Among the remaining effectors suggestive of HGT, six were found on scaffolds with high identity to *F. oxysporum* which were mostly absent from *F. verticillioides* too, consistent with transfer of a larger region of DNA, whereas five were assembled into a background of low identity, consistent with transfer of a shorter piece of DNA (Additional file 2: Figures S8 and S9).

### Evidence that class II TEs play a role in horizontal transfer of putative effectors

**Fig. 9:**
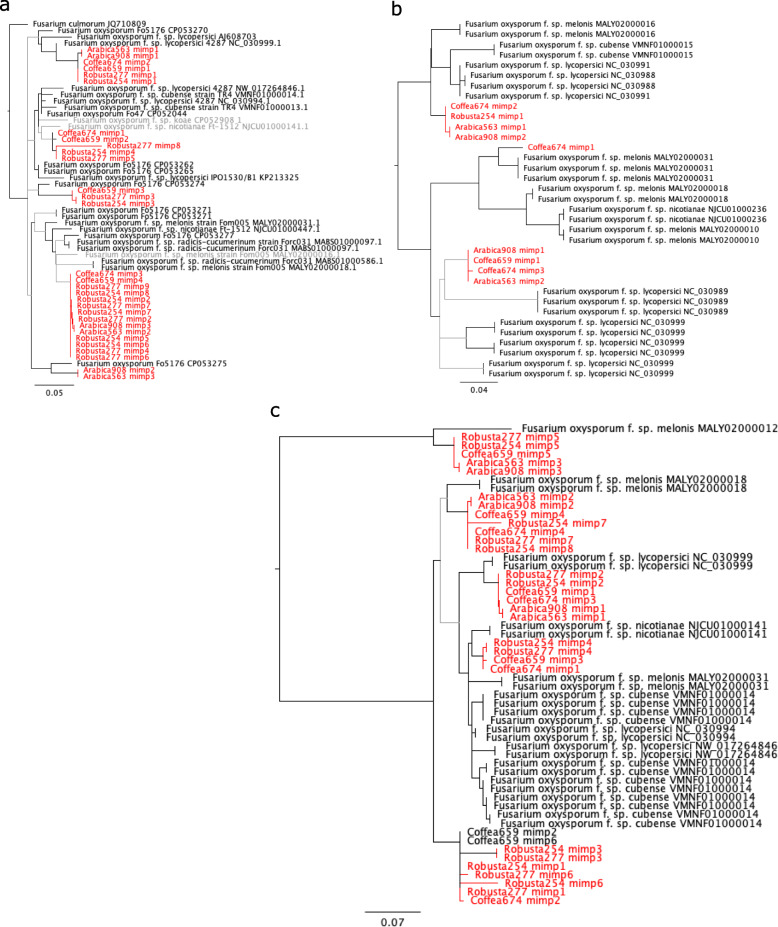
*mimp* families found in *F. xylarioides*. Phylogeny of three *mimp* families in *F. oxysporum, F. xylarioides* and *F. culmorum* (tree rooted with *F. culmorum* in a). **a**, *mimp* family 1. **b**, *mimp* family 4. **c**, *mimp* family mn4. No other species matches were returned compared to the species in Additional file 2: Table S3 and the nr database (BLAST, 1e-50). Nodes which are coloured in black share a branch support value >90%, *F. xylarioides mimps* are annotated in red. Phylogeny was inferred using Chi2 support values. Drawn in Geneious 9.1

Previous work implicated *mimp* class II TEs in the HGT of effector genes in *F. oxysporum*: *mimp*s are strongly associated with effector genes [13] and phylogenetic analysis support multiple transfer events between *F. oxysporum* and other FFC species [62]. We therefore investigated the potential role of *mimp*s (Additional file 2: Table S6) in horizontal acquisition of effector genes in *F. xylarioides*. We find 46 *mimps* on average per genome, with 59 in the robusta and *Coffea* strains and 20 in the arabicas, which is at the upper end of estimates in other FFC species [62] (<20 in each strain except for *F. hostae* which has 40), despite a potential bias to under-estimate numbers of repetitive TEs in assemblies of short-read data (section 3.1). Most are from the common *mimp* 1 and 2 families, with 10% in family 4 in the robusta and *Coffea* strains and 10% in the highly divergent and recently described *mimp* family *mn14* in the arabicas (Fig. 9). We also found at least one full, and therefore presumably active, copy of the FOM24 *impala* transposase ORF (BLAST, e=0.00) in each strain. To test whether *F. oxysporum* could be the source of these *mimps*, we compared nucleotide sequences. Using BLAST, we compared the *mimps* to the nr database as well as the genomes in Additional file 2: Table S3, and found only hits in *F. oxysporum*. We found *mimps* in families 1, 4 and the highly divergent family mn4 to have >96% percent identity (>97% in 1 and mn4) with *F. oxysporum* with high support values (Fig. 9) and the *F. xylarioides* and *F. oxysporum mimps* as sister clades.

Of the 15 cases of putative horizontal transfer discovered by phylogenetic analysis of effector gene sequences in the previous section, all are found on scaffolds containing *mimps* and DNA transposons. For example, the 20Kb scaffold from Fig. 8e, which is present in the robusta and *Coffea* genomes, has close affinity to *F. oxysporum* and is enriched and interspersed with class II DNA transposons and *mimps*. This region matches a chromosomal mini-cluster on *Fol*’s chromosome 14 that contains SIX effectors (being within 1.8kb to 33kb of SIX genes, Additional file 2: Figure S6, [13]). [13] suggested that effector genes found between interspersed repeat regions on chromosome 14 could coincidentally be transposed with the *mimps*, which provides a working hypothesis for how *F. xylarioides* acquired these effectors. In addition, three more putative effectors match to mobile chromosomes that have not been linked with pathogenicity: *six10* and *pep1* match regions of chromosomes 3, 6 and 15, while *og14180* matches 3 and 6.

Beyond these examples, our full set of 64 effector loci tend to be close to TEs: the median distance from a TE for our putative effectors ranges from 2.2kb to 3.9kb, compared to 4.4kb to 7.6kb on average for random subsets of genes chosen from the whole genome (randomisation test, p <0.05 in robusta277, *Coffea674* and arabica563, p >0.05 in the other genomes, Additional file 2: Table S7). These numbers might be underestimates of average distances for effector genes found in lineage specific regions that are rich in repeats and TEs that we were unable to assemble. However, not all of the effectors associated with class II transposons display a signal of HGT in our phylogenetic analysis of effector genes. Another possible explanation for the association is that TEs and effector genes cohabit genome islands with high rates of variability, perhaps with TEs directly contributing to increased variability or modified expression of effector genes. In potential support of this idea, the effector genes from the reference-guided scaffold assemblies tend to be close to LRAR-affected regions targeted heavily by RIP-mutation (section 3.1, median distance from LRAR-affected regions ranges from 6.4kb to 22.5kb, compared to 17.5kb to 28kb for random subsets of the same number of genes from the whole proteome, p <0.05 in robusta254, arabica563, and arabica908, p >0.05 in the other genomes,). RIP-mutation and TE transposition within genomes might therefore provide additional mechanisms for enhancing variability of the effector genes [11], which could be explored by further analyses of population variation.

## Discussion

Using fungal strains collected over a period of 52 years and optimally cryopreserved in a living state, we uncovered the genomic basis of repeated outbreaks of *F. xylarioides* on different commercial coffee species. Our data support the conclusion that the robusta and arabica populations had divergent origins within the *F. xylarioides* clade, with the robusta population deriving from within the earlier outbreaks of more genetically diverse strains on alternative *Coffea* crops. Furthermore, by cataloguing multiple putative effector and pathogenicity genes, we show how the different host-specific populations diverged in gene content and sequence by vertical processes within lineages, as well as by apparently acquiring 15 putative effector genes by horizontal gene transfer, mostly in the robusta and *Coffea* strains, from *F. oxysporum*.

The finding that arabica is divergent from the other strains is consistent with earlier work based on crossing experiments and molecular markers, which supports the reclassification of the 1990-2000s host-specific populations as separate species named *F. abyssiniae* (arabica) and *F. congoensis* (robusta). Our evidence for concordant gene trees across the entire genome, as well as substantial differences in gene content, both overall and specifically for genes thought to be important for the infection process, lends weight to this proposal. Rather than the presumed recent emergence of these strains, the level of diversity we observe is consistent with a divergence far earlier than the emergence within the last 50 years of these populations as major disease agents. Moreover, we found little evidence for transfer of genes from the pre-1970s outbreak into the 1990-2000s epidemic on arabica coffee. We hypothesise that this represents a separate emergence on commercial arabica coffee from *F. xylarioides* strains associated with wild coffee relatives in Ethiopia or previously less noticeable disease symptoms on arabica coffee, but further sampling, including of wild relatives, would be needed to test this. In contrast, the robusta population evolved with relatively minor modifications from the original outbreak on multiple *Coffea species* in central and west Africa, with only two putative effector genes gained and a handful lost compared with the *Coffea* strains sampled here. The high level of similarity between paired strains within each host-specific population, despite sampling from different locations or countries five years apart, supports the emergence of each population from low initial diversity, i.e. each constitutes a single epidemic outbreak replacing earlier variants [29, 71].

The observation that 15 effector genes and their flanking regions are class II TE- and *mimp*-enriched, share a high percent identity with *F. oxysporum* and have emerged nested within *F. oxysporum* homologs, supports the role of horizontal transfer in the origin of host-specific differences. The mobile pathogenic chromosome of *F. oxysporum* is widely reported to transfer pathogenicity between different strains [9, 13]. However, to our knowledge, the transfer of pathogenicity has not been reported across different *Fusarium* species, nor has it been linked with the other mobile chromosomes. *F. oxysporum* is known to undergo conidial anastomosis [72], so such transposition could have taken place in their shared niche, where both *F. xylarioides* and *F. oxysporum* (undescribed f. sp.) have been co-isolated from the roots and wood of CWD-infected coffee in Ethiopia and central Africa, as well as from banana roots where banana and coffee were intercropped in Uganda [29, 41]. *F. oxysporum* f. sp. *cubense* (*Foc*) is widespread on banana in east and central Africa [41]. *F. xylarioides* is also known to infect tomato fruits [73] and cotton seedlings [74], offering potential alternate hosts. While [75] did not find *F. xylarioides* in 105 different crop and weed species collected surrounding coffee farms in Uganda, the ability of *F. xylarioides* to infect solanaceous crops and the Kayinja banana cultivar were investigated [71] (no findings published). Therefore, *Fol, Foc* and *F. oxysporum* f. sp. *vasinfectum* (cotton) are of interest, as well as other *formae speciales* which are the closest match to numerous effectors: *F. oxysporum* f. sp. *pisi* and *F. oxysporum* f. sp. *raphani*. If confirmed, this would suggest avoiding the widespread practice of intercropping species that share closely related pathogens, for example coffee and banana, or weed management for those which share pathogens, for example of solanaceous weeds in crop surroundings. Greater sampling from these taxa and around coffee fields would be needed to further clarify the range of potential donors for horizontally acquired genes.

Contrary to previously described cases in *F. oxysporum* [9, 13], we find no evidence that transfer occurred on a known mobile chromosome, and indeed many of our putative effector genes are spread across our genome scaffolds. It remains possible that an unknown mobile chromosome or chromosomes with some homology to the *Fol* mobile chromosomes is involved with some of the variation among strains that we observe: we were unable to assemble LS and FXU regions into whole chromosomes, where matches to *Fol* mobile chromosomes were concentrated. Future long-read sequencing allied to greater sampling of co-occurring taxa would confirm or refute this possibility.

Our results also provide the first insights into the potential genetic basis for pathogenicity in coffee wilt disease. Of 64 putative effector genes we identified, roughly one third were shared by all *F. xylarioides* isolates. These are expected to include genes that contribute to the common pathogenicity traits of these fungi, i.e. those effectors which migrate to and induce vascular wilting first in the main infected stem and latterly in the systemic infection of the coffee tree as visualised as premature ripening of coffee berries [20]. The main differences that we detected between host-specific populations were in gene content and expansion of gene families. For example, while *Fusarium* is broadly enriched for lytic enzymes involved in carbohydrate metabolism [55, 56], and vascular wilt inducers for certain gene families (as also reported by [10]), we found differences between the arabica and robusta populations, particularly in enzymes involved with metabolism of plant cell wall components. Specifically, the host-specific populations had different enzymes although they share similar functions in the hydrolysis of chitins, pectins, xylans, mannose and rhamnose. For example the arabica strains share seven CAZyme copies, whereas the robusta strains share 19 different CAZymes with the *Coffea* strains that are involved in the breakdown of the same sugars, as well as cellulose (none are found just in the robusta strains). Whilst we cannot precisely infer the phenotypic effects of these changes, expanded cell-wall degrading enzymes imply variation in the capacity or mode of *F. xylarioides* to breakdown plant cell walls. Cell-wall breakdown releases pectins into the xylem vessels, which could unintentionally act as a barrier to further pathogen growth, so pectin-degrading enzymes could break down pectin barriers for the pathogen to spread leading to the external symptoms of wilting as infection progresses [36]. The ability to utilise plant carbon suggests the fungus is able to live within host coffee plants for a long time before the disease state becomes evident [10], something which has been reported from the field for *F. xylarioides* [20]. A key benefit of the availability of living samples is that we can now begin to test the importance of these differences with functional assays.

### Conclusion

Our results highlight the multigenic nature of how fungi respond to changing crops and host specialisation. The complexity of the process whereby new effectors emerge means that many pathogenicity genes have functional redundancy, whereby disruption of single genes does not affect virulence [76]. Therefore, it is expected that no single gene confers host specificity, but a diverse set of pathogenicity genes is required. In turn, the emergence of newly pathogenic or host-specific strains involves shifts in profiles of multiple genes. This can occur by transfer of whole pathogenicity regions or from the emergence of distinct lineages that diverged over longer periods of time, but later independently emerge as significant crop pathogens, as observed here for the arabica versus robusta populations. Our finding of the transfer of pathogenicity factors between more distantly related taxa raises a particular concern for the currently widespread practice of intercropping plant species which share closely related plant pathogens, which might increase the probability of new virulent pathogens emerging. These findings prove the value of culture collections in providing historic and optimally preserved strains which help us to understand and model the evolution and spread of disease.

## Methods

### Strain information

Six *F. xylarioides* strains were selected for Illumina MiSeq sequencing (Table 1). All strains were originally collected over a 52 year period from CWD-infected trees and stored in the CABI culture collection (CABI, Egham, Surrey, TW20 9TY). Strains were grown on synthetic low nutrient agar (SNA) at 25^∘^C, and potato sucrose agar (PSA) with UV irradiation in order to confirm morphological identification following [30]. Following [77], strains were grown in GYM broth and genomic DNA was extracted from <20mg of washed mycelium, which was frozen in liquid nitrogen before extraction folllowing the DNeasy Plant Mini Kit (Qiagen, Hilden, Germany) standard protocol.

### Genome sequencing and assembly

For each strain, a single library was prepared with the Illumina TruSeq PCR-free kit and sequenced with the Illumina MiSeq v3 600 cycle kit with 2 x 300 bp paired end sequencing and 350bp insert size at the Department of Biochemistry (University of Cambridge, UK). Low quality bases and adaptors were identified and trimmed (quality score <20) using TrimGalore 0.6.0 by Cutadapt (https://github.com/FelixKrueger/TrimGalore, [78]). Overlapping paired-end reads were merged using FLASH 1.2.11 [79] before being assembled using MEGAHIT 1.2.8 [44] with a final k-mer length of 189. Assembly metrics were computed using QUAST 5.0.2 [80] (Table 1 and Additional file 2: Table S1). The quality of the genomes in terms of the presence of 303 core eukaryotic genes and 290 core fungal genes was assessed using BUSCO v3.0.2 [81]. All raw sequence data and assembled genomes have been deposited in the relevant International Nucleotide Sequence Database Collaboration (INSDC) databases under the BioProject accession number PRJNA659227 (see Additional file 2: Table S9 for SRA run accessions). For comparison, we selected eleven genomes of related species (Additional file 2: Table S3) selected for being: closely related and wilt-inducers (*F. udum, F. oxysporum f. sp. lycopersici* and *f. sp. cubense*); closely related which occupy the same coffee-plant niche (*F. verticillioides, F. solani* and *F. oxysporum*); closely related (*F. fujikuroi, F. mangiferae, F. proliferatum*); and wilt-inducers (*V. dahliae* and *V. albo-atrum*). Chromosomal and lineage-specific regions in each *F. xylarioides* assembly were identified by whole-genome alignment to the chromosomal level assembly of *F. verticillioides* (GenBank accession GCA_003316975.2, Ma et al. 2010). The *Coffea659* assembly (chosen for having the larger genome of the two *Coffea* strains) was aligned against *F. verticillioides* using RaGOO v1.1 with the ‘-C’ parameter [82], with the remaining *F. xylarioides* strains aligned in-turn against the assembled RaGOO *Coffea659*. We used BLAST (with 1.00e-50) to identify scaffold specific groups between the closely related *F. xylarioides, F. udum* and *F. verticillioides*: scaffolds which did not match to *F. verticillioides* but are present in each *F. xylarioides* strain and the *F. udum* genome (GCA_002194535.1, Srivastava 2018) were interpreted as *Fusarium xylarioides* and *udum*-specific (FXU). Scaffolds which are present only in *F. xylarioides* were interpreted as *F. xylarioides*-specific (FXS), while scaffolds which did not match to the historic *Coffea659* genome nor the *F. verticillioides* assembly nor *F. udum* were concluded to be lineage-specific (LS). Here, the LS scaffolds relate to the host-specific populations.

### Gene prediction and orthologous clustering

Protein-encoding genes were predicted using the BRAKER v2.1.2 pipeline [83–88], using RNA-seq data from the closely related *F. verticillioides* to guide gene models. RNA-seq reads (SRR10097610) were mapped to each genome using STAR v2.7.3a [89] and the resultant BAM file was input to BRAKER with default settings. BUSCO analysis was performed to assess quality of predicted proteomes, and the GC content of coding sequences was calculated. This was used to calculate GC-equilibrated regions in 20 kb windows, where GC-rich blocks have a GC content within 1% of that of the coding sequences, as well as AT-rich blocks, where the GC content is <= 0.7 of the coding sequences content [11]. A Kruskal-Wallis chi-squared test confirmed association between genes and the GC-rich blocks, with a Wilcoxon rank test (Bonferroni adjustment) to confirm significance between pairs. Three programs were used to infer functional classification: Interproscan v5.35-74.0 [90]; Superfocus [91] which classifies the proteins by their SEED categories [92]; and NCBI BLAST (https://blast.ncbi.nlm.nih.gov/Blast.cgi) using megaBLAST searches and the nr database. All proteins lacking significant hits were annotated as hypothetical proteins. OrthoFinder v2.3.8 [93, 94] was used to determine orthologous groups amongst the six *F. xylarioides* strains and eleven related species (Additional file 2: Table S3 and Additional file 1). Orthofinder also reconstructed a species tree from gene trees of the entire set of orthologs reconstructed by FastML (Fig. 4). Concordance of the set of individual gene trees for single ortholog groups was summarised using PhyParts (https://bitbucket.org/blackrim/phyparts/src/master/), while concordance of the ortholog groups was summarised using venn diagrams drawn with the Venn package in RStudio. Correlations between strains were tested for an excess of sharing using the “SuperExactTest” package in RStudio ([95].

### Transposable elements and repeat annotation

Repeats and transposable elements (TEs) were identified directly from the assembled nucleotides. RepeatModeler v2.0 was run with parameters “-LTRStruct -pa 32” to first construct a custom repeat library for each genome [96]. This library was then used to call TEs and other repeats from the genome scaffolds using RepeatMasker v4.1.0 with the parameters “-pa 32 -lib $LIB -dir. -alignments -gff -no_is” [97]. The distribution of TEs across the genome was represented as the fraction of base pairs within 20kb windows assigned to TEs. In the same way as for genes, the association between TEs and AT-rich areas was tested using a Kruskal-Wallis chi-squared and a Wilcoxon rank test (bonferroni adjustment). The separate distribution of Class 1 RNA retrotransposons and Class 2 DNA transposons [98] was also calculated. For comparison, repeats and TEs were also identified from the raw reads using DNAPipeTE [99] for our *F. xylarioides* strains, as well as *Fol* (SRA Accession SRR3142258).

The presence of repeat-induced point mutations (RIP) were confirmed using The RIPper bioinformatics tool [49], with default settings applied to identify Large RIP Affected Regions (LRARs), and the RIPCAL [100] RIP-index scan with default threshold settings applied and a scanning subsequence length of 10kb for the core chromosome scaffolds and 300bp for the FXU and LS scaffolds to reflect the average sequence length across the different scaffold groups.

### Searching for putative effector genes involved in wilt disease

We adopted a four-pronged approach to search for putative effector genes involved in the host-specificity between the arabica and robusta populations. Only single gene copy orthologous groups were analysed for ease of comparison. We used BLAST to verify inferred patterns of presence and absence based on the orthogroups, and corrected in the twelve cases (annotated with an “a” in Fig. 6) where annotation had missed a full length copy of the gene in certain taxa.

**Pre-characterised effectors** Putative effector sequences described in closely related *Fusarium* species were downloaded from Genbank (Additional file 2: Table S3). Using BLAST, the sequences were searched for against the *F. xylarioides* predicted genes and scaffolds. Proteins were judged to be present if a blast match of >70% and 1.00E-50 was obtained and the sequence retrieved from the genome scaffold, checking to include the full protein region if the blast match only returned a partial match.

**Small, cysteine-rich putative effectors** Many fungal effector proteins are small (<400 amino acids), cysteine-rich (>4 cysteine residues) and secreted [8, 52]. Putative effectors were therefore searched for within each genome based on size, cysteine-richness, secretion signal and putative function. First, annotated genes were sorted according to size and number of cysteine residues using SeqKit [101], to select those with <400 amino acids and >4 cysteine residues. Next, the presence of a signal peptide and a signal peptide cleavage site on those proteins were predicted using TargetP 1.01 [102, 103] with an RC score cut-off 1-3 to increase specificity. Finally, subcellular localizations for these proteins were predicted using WoLF PSORT (https://wolfpsort.hgc.jp/) to identify secreted extra-cellular proteins. The orthologous gene sets for those genes were identified from the Orthofinder results.

**CAZyme effectors** CAZymes are carbohydrate-active enzymes thought to be important in the infection pathway. Carbohydrates in plant cell walls provide the main source of carbon for fungal pathogens [54] and consist of cellulose microfibrils embedded in a matrix of hemicelluloses, pectin polysaccharides and glycoproteins [104]. The breakdown of cell wall polymers requires a range of enzymes, including glycoside hydrolases (GHs), pectate and polysaccharide lyases and carbohydrate esterases (CEs) [34]. CAZymes that target cell walls also contain carbohydrate-binding modules (CBMs) [105], which bind to cell wall polymers and increase the enzymes’ catalytic efficiency by improving contact [54, 106]. CBMs show specificity for particular polymers [107]. Broadly, *Fusarium* is enriched for CAZymes (Additional file 2: Table S5). Therefore, to identify putative CAZyme-encoding effector genes, and the CBM and their associated carbohydrate active modules differentially expressed across the wilt-inducing and non-wilt inducing fungal strains we used the CAZy database (www.cazy.org, [67]) to identify CAZyme-encoding orthologous groups. We looked for those present in only the wilt-inducing strains using BLAST (e-vale 1.00e-50), additionally allowing for those which were present in just one non-wilt inducing strain.

**Effector genes with TEs in their promoter regions** In particular, class 2 MITES (Miniature Inverted-repeat Transposable Elements) have been shown to associate with effector proteins [13]. The abundance of class II transposons recognised by RepeatModeler, miniature and full impala transposons, as well as recently described transposons across the genomes was tested using the accessions detailed in Additional file 2: Table S6 and by [61], with a BLAST (score >70%, 1.00e-50, length >150bp) confirming their presence. The presence of an intact impala ORF was confirmed with the intact FOM24 transposase (Genbank accession AF282722.1) using BLAST, 1e-50.

For each set of putative effectors, nucleotide sequences were aligned using MAFFT and maximum likelihood trees reconstructed using PhyML 3.0 [108] with a Generalised Time-Reversible model with invariant sites and gamma distributed variation in substitution rates across 4 rate classes of sites, implemented in Geneious v9.1. We also located each putative effector on the genome scaffolds in each species mapped against the chromosomal level *F. verticillioides* assembly. The site test of positive selection in PAML v4.8 [109] was used to test for positive selection among codons in the DNA sequences in each phylogenetic tree by estimating the ratio between synonymous and nonsynonymous substitutions (*ω*). PAML detects positive selection when is significantly greater than 1 for a subset of codons. We compared a null model of “NearlyNeutral” selection that includes a class of codons under purifying selection and a class that evolve neutrally, against an alternative model of “PositiveSelection” that additionally includes a class with >1. Log-likehood ratios were calculated and compared with a chi-squared test with one degree of freedom to confirm significance. A Bayes Empirical Bayes (BEB) approach was used to identify specific amino acid sites under positive selection within gene sequences which were confirmed to be under positive selection [110]. We detected horizontal gene transfer by searching for our 64 putative effector genes across the genomes in Additional file 2: Table S3 and *F. oxysporum* forma speciales in Additional file 2: Figure S7. BLAST hits were aligned using MAFFT and maximum likelihood trees reconstructed using approximate Likelihood Ratio Tests >0.95. We then followed the decision tree (Fig. 7) to decide if each gene displayed evidence of horizontal gene transfer: is the effector present in other FFC species (Y / N); if Y, does *F. xylarioides (Fx)* nest with the FFC gene copies (Y / N); if N to either of previous decisions, does Fx nest with *F. oxysporum (Fo)* thus disrupting the Fo phylogeny (Y / N); branch support values (BSV) for Fx with Fo (n); if N, does Fx nest with Fo by distance i.e. less distance from Fx to Fo than greatest distance from Fo to Fo; pairwise id % for whole branch with support value for Fx nested with Fo.

## Supplementary Information

**Additional file 1** Orthogroups. Output from Orthofinder v2.3.8 describing the orthologous groups and genes amongst the six *F. xylarioides* strains and eleven related species.

**Additional file 2** Supplemental figures and tables.

## Data Availability

The genomic sequences of *F. xylarioides* have been deposited at Genbank under the BioProject accession number PRJNA659227. Whole genome data generated in this study has been deposited at the Sequence Read Archive under the following SRA accession numbers: SRR12534416; SRR12534415; SRR12534414; SRR12534413; SRR12534412 and SRR12534411. Further details are in Additional file 2: Table S9. The genome assemblies for *F. verticillioides* and *F. oxysporum* f. sp. *lycopersici* under accession numbers GCA_003316975.2 and GCA_000149955.2 were used in reference-guided scaffolding to align our *F. xylarioides* scaffolds to their assembled chromosomes. Additional RNA-seq data used in annotation of *F. xylarioides* and *F. udum* genomes were obtained from *F. vertcillioides* under accession number SRR10097610. Proteome data for 10 *Fusarium* and *Verticillium* fungal species used for phylogenetic orthology inference were obtained from Genbank using the following BioProject accessions: *F. verticillioides*, PRJNA245136; *F. fujikuroi*, PRJNA322155; *F. mangiferae*, PRJEB9887; *F. proliferatum*, PRJNA576857; *F. oxysporum* f. sp. *lycopersici*, PRJNA342688; and *F. oxysporum* f. sp. *cubense*, PRJNA529756; *F. solani*, PRJNA16586; *F. graminearum*, PRJNA243, *V. dahliae*, PRJNA225532; and *V. albo-atrum*, PRJNA51263. Recently published genome sequence data for two additional *F. xylarioides* strains were used for comparison and verification of genome statistics. The data was obtained from Genbank genome database under accession numbers PRJNA530275 and PRJNA508603. The individual gene sequences used in this study were all obtained from the Genbank database. The sequence data for the 17 known effector genes from fungal plant pathogens used to identify our first class of putative effector genes (section 3.3.1) are described in Additional file 2: Table S4. Additionally, a pathogenic *pda1-a* allele from sugarbeet (Genbank accession number AY487143.1) was used for comparison to the *pda1* gene identified in this study. The sequence data for the *impala* and *miniature impala* and newly-described class II transposable elements is described in Additional file 2: Table S6, detailing the Genbank accession number, the type of transposon and whether the sequence is partial or complete. Twelve of these transposable elements were not obtained from Genbank, but were obtained from [13], with the reference detailed in Additional file 2: Table S6. The FOM24 transposase, Genbank accession AF282722.1, was used to confirm the presence of an intact impala ORF.

## Abbreviations

ISNDC: International Nucleotide Sequence Database Collaboration
f. sp.: formae speciales
BLAST: Basic Local Alignment Search Tool
TE: Transposable Element
CWD: Democratic Republic of Congo
CABI-IMI: CABI International Mycological Institute
SIX: Secreted In Xylem
BUSCO: Benchmarking Universal Single-Copy Orthologs
FFC / GFC: *Fusarium fujikuroi* complex / *Gibberella fujikuroi* complex
Fol: *Fusarium oxysporum* f. sp. *lycopersici*
Fv: *Fusarium verticillioides*
FXU: *Fusarium xylarioides* and *-udum* specific
FXS: *Fusarium xylarioides*-specific
LS: Lineage specific
RIP: Repeat Induced Point-like
LRAR: Large RIP (Repeat Induced Point-like) Affected Regions
Pfam: Protein families
CAZyme: Carbohydrate-active enzyme
CBM: Carbohydrate-binding module
GH: Glycoside hydrolase
MITE: Miniature inverted-repeat transposable element
mimp: Miniature *impala*
CE: Carbohydrate esterase
LTR: Long terminal repeat
Foc: *Fusarium oxysporum* f. sp. *cubense*
PL: Polysaccharide lyase
HGT: Horizontal gene transfer
ORF: Open Reading Frame
Fom: *Fusarium oxysporum* f. sp. *melonis*
SNA: Synthetic low nutrient agar
BSV: Branch support values
