# Correction to: Historical genomics reveals the evolutionary mechanisms behind multiple outbreaks of the host-specific coffee wilt pathogen *Fusarium xylarioides*

**DOI:** 10.1186/s12864-021-07831-8

**Published:** 2021-07-07

**Authors:** Lily D. Peck, Reuben W. Nowell, Julie Flood, Matthew J. Ryan, Timothy G. Barraclough

**Affiliations:** 1 Science and Solutions for a Changing Planet Doctoral Training Partnership, Grantham Institute, Imperial College London, South Kensington, London, SW7 2AZ UK; 2 Department of Life Sciences, Imperial College London, Silwood Park Campus, Ascot, Berkshire, SL5 7PY UK; 3 Department of Zoology, University of Oxford, 11a Mansfield Road, Oxford, OX1 3SZ UK; 4 CABI, Bakeham Lane, Egham, Surrey, TW20 9TY UK

**Correction to: BMC Genomics 22, 404 (2021)**

**https://doi.org/10.1186/s12864-021-07700-4**

Following publication of the original article [1], it was reported that Matthew J. Ryan’s name was erroneously published as Matthew R. Ryan.

The original article has been corrected.
