# Supplementary material for: Historical genomics reveals the evolutionary mechanisms behind multiple outbreaks of the host-specific coffee wilt pathogen Fusarium xylarioides
Source: BMC Genomics. 2021 Jun 4;22:404. doi: 10.1186/s12864-021-07700-4 (PMC8176585; doi:10.1186/s12864-021-07700-4)
Supplement: Supplementary file 2 — Additional file 2 Supplemental figures and tables. [file 12864_2021_7700_MOESM2_ESM.pdf]

1. SUPPLEMENTARY MATERIAL

A

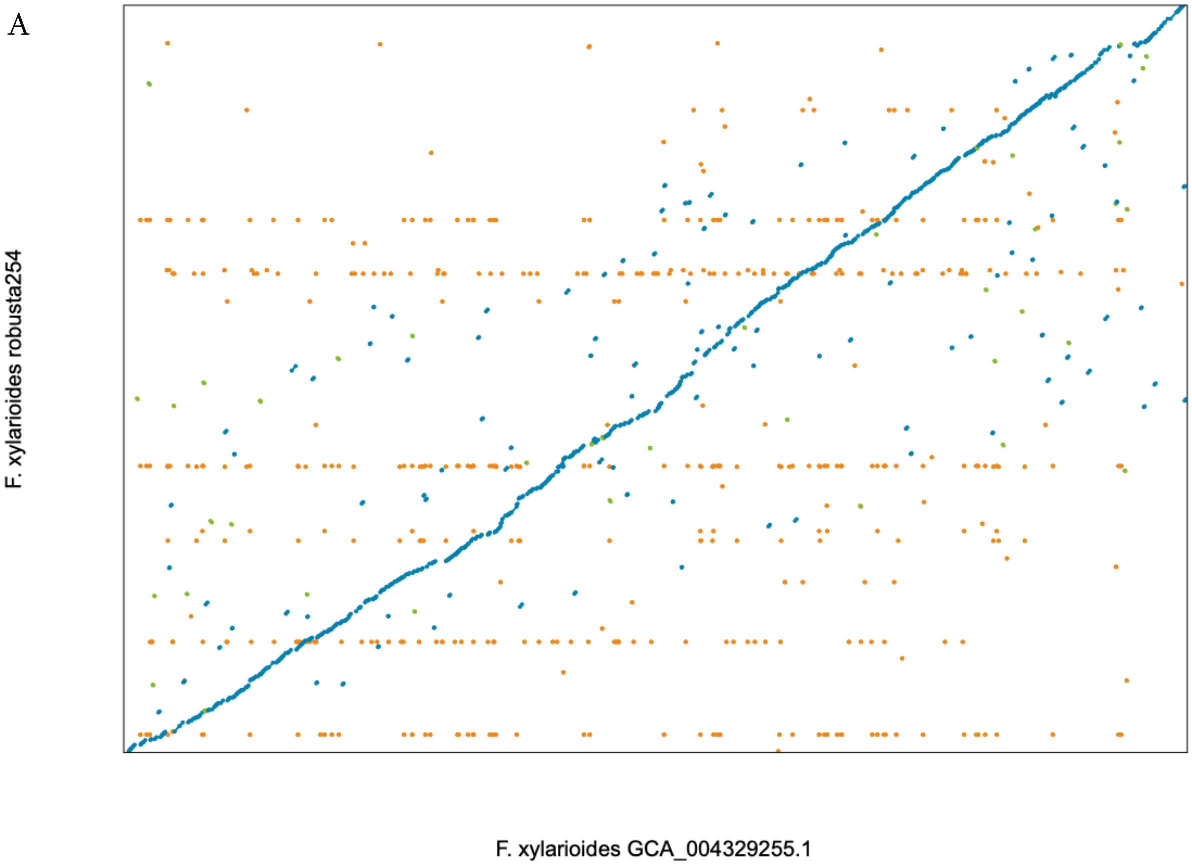

Fig. S1. Representative whole-genome alignments of *F. xylarioides* robusta strains sequenced in this study with publically available *F. xylarioides* robusta strains. Each dot represents chromosomal correspondence, with absences representing absent chromosomes. Genomes were aligned using nucmer in the MUMmer3 package, with outputs processed using DotPrep.py and visualised using Dot in DNANexus. Blue indicates forward alignments, green indicates reverse alignments, orange indicates repetitive alignments.

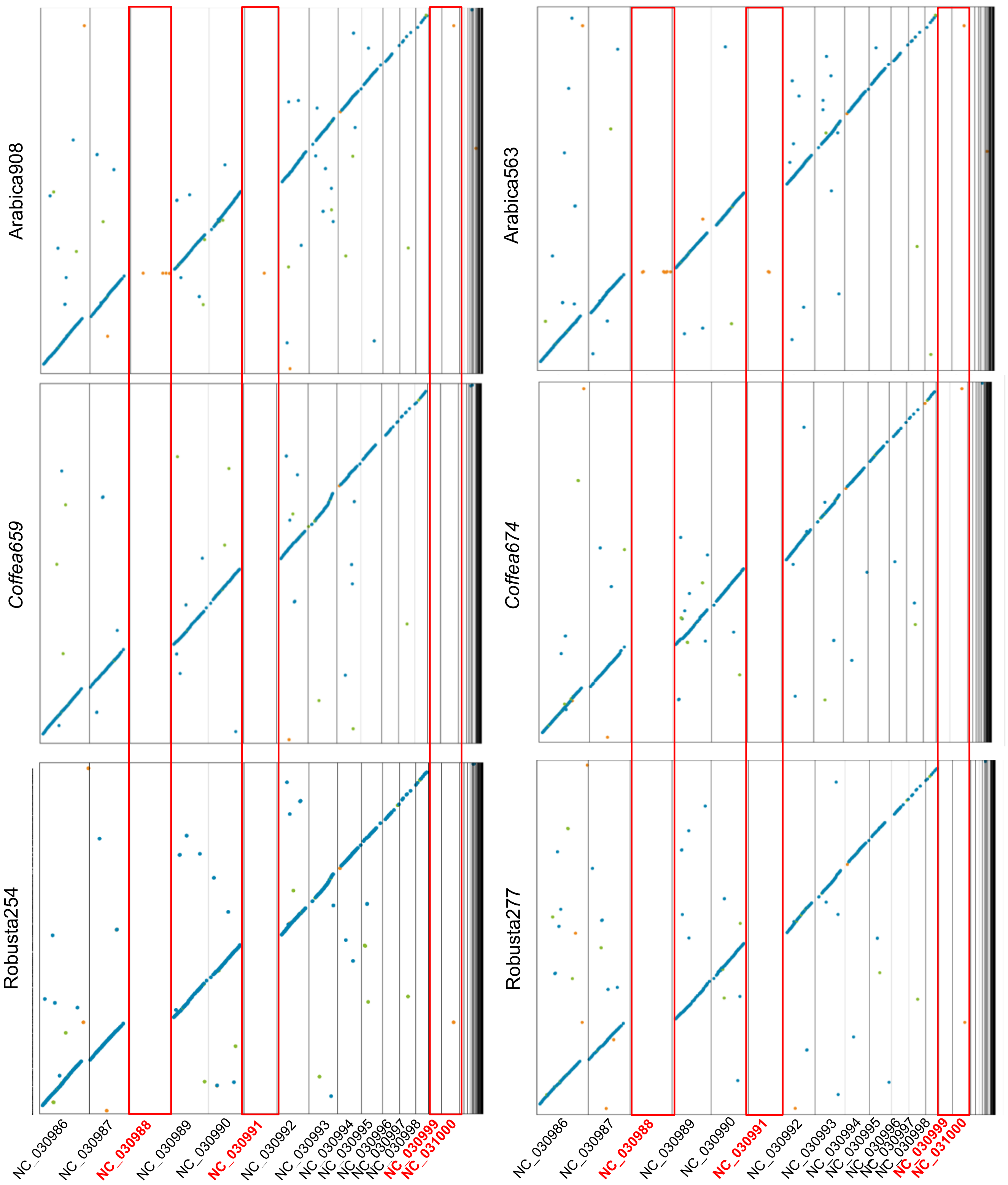

Fig. S2. Representative whole-genome alignments of *F. xylarioides* strains against the 15 *F. oxysporum f. sp. lycopersici* (*Fol*) chromosomes. Each *Fol* chromosome is labelled, with the four fully mobile chromosomes annotated in red: chromosomes 3, 6, 14 and 15. The remaining 11 chromosomes are the syntenic, core chromosomes shared with sister *Fusarium* species. Each dot represents chromosomal correspondence, with absences representing absent chromosomes. Genomes were aligned using nucmer in the MUMmer3 package, with outputs processed using DotPrep.py and visualised using Dot in DNANexus. Blue indicates forward alignments, green indicates reverse alignments, orange indicates repetitive alignments.

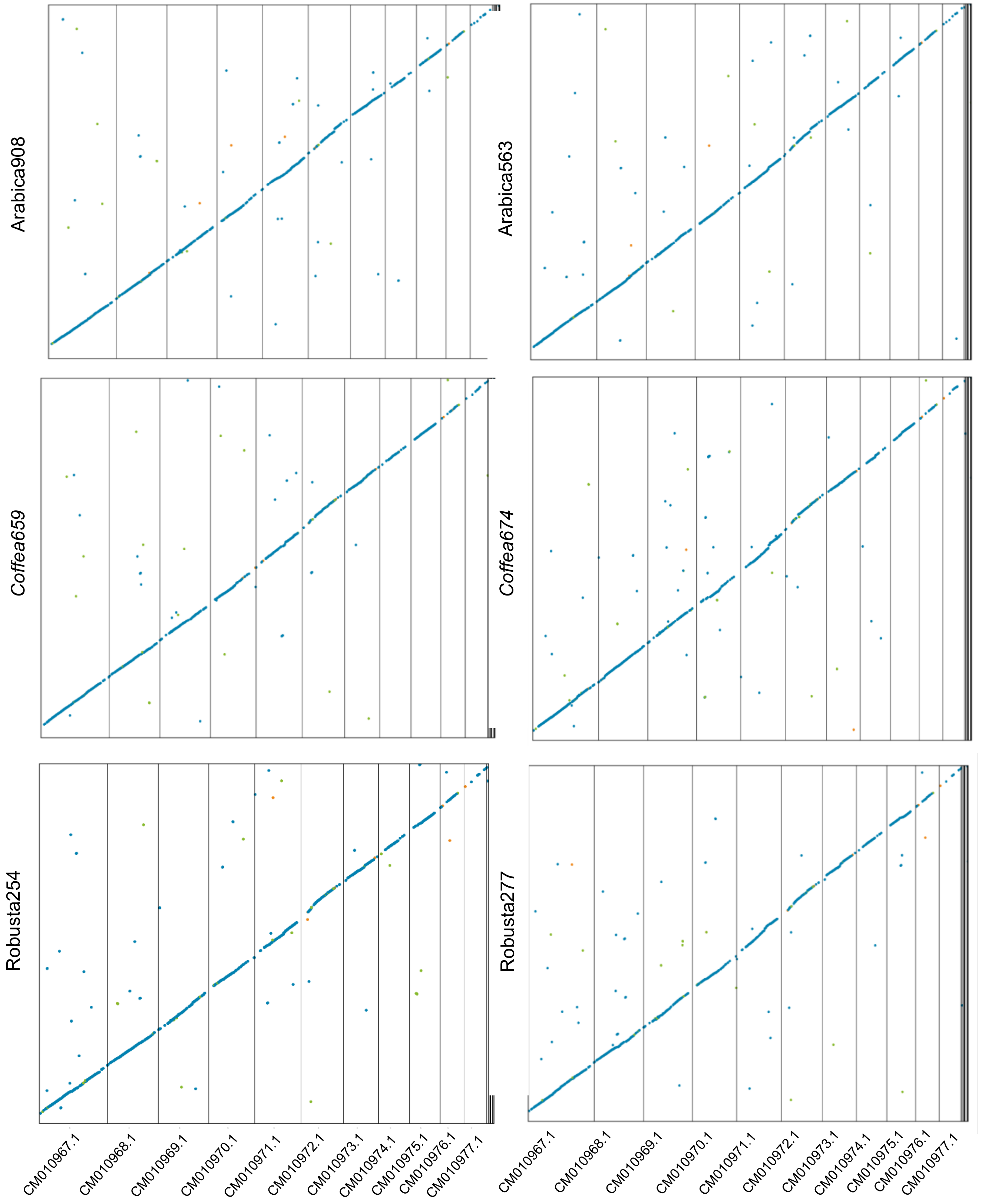

Fig. S3. Representative whole-genome alignments of *F. xylarioides* strains against the 11 *F. verticillioides* syntenic, core chromosomes shared with sister *Fusarium* species. Each dot represents chromosomal correspondence, with absences representing absent chromosomes. Genomes were aligned using nucmer in the MUMmer3 package, with outputs processed using DotPrep.py and visualised using Dot in DNANexus. Blue indicates forward alignments, green indicates reverse alignments, orange indicates repetitive alignments.

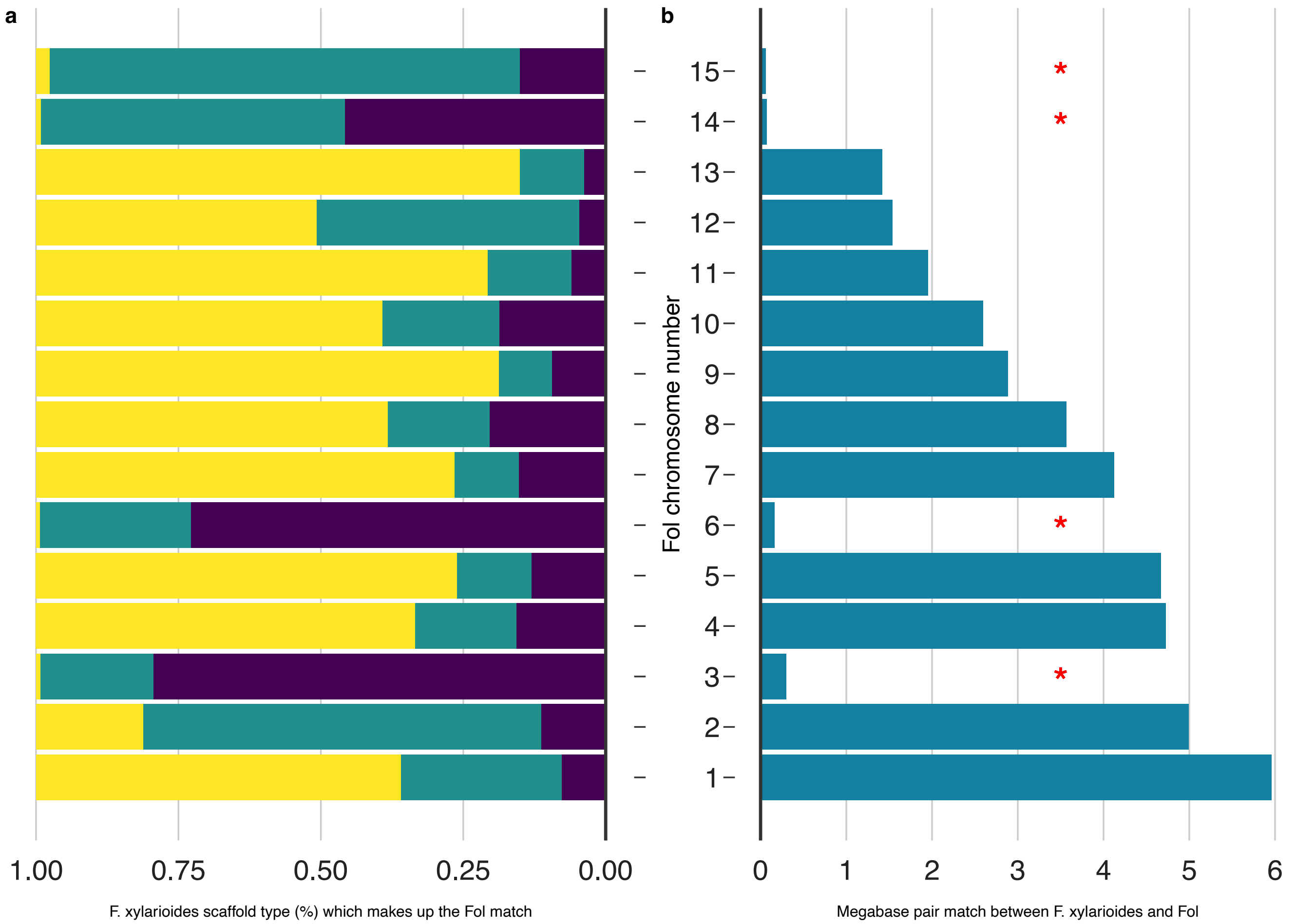

Fig. S4. *F. xylarioides* scaffolds mapped to *F. oxysporum* f. sp. *lycopersici* (*Fol*) chromosomes using reference-guided scaffolding. **A** The type of *F. xylarioides* scaffold group which match each *Fol* chromosome: yellow, scaffolds which match to core chromosomes in the *F. xylarioides* assembly mapped to *F. verticillioides* (FV); green, scaffolds which match the *F. xylarioides*- and *F. udum* (FXU) specific scaffolds (i.e. shared by these species and absent in *F. verticllioides*); purple, scaffolds which match the Lineage Specific (LS) scaffolds and are not shared across the *F. xylarioides* strains. **B** The total megabase pair match between *F. xylarioides* scaffolds and *Fol* chromosomes, with mobile chromosomes annotated with a red asterisk.

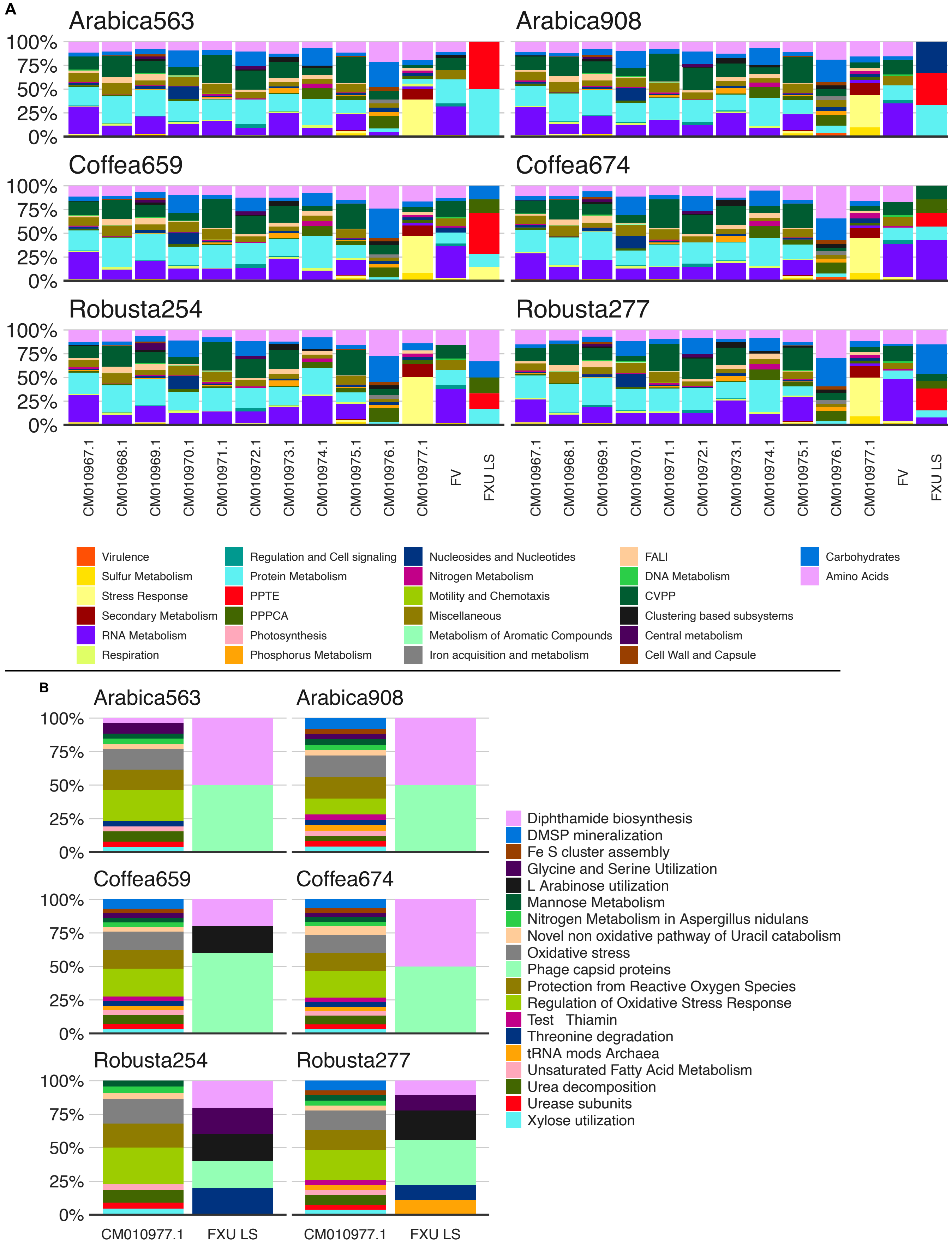

Fig. S5. Functional diversity of *F. xylarioides* across the core chromosomes and by scaffold group: FV, scaffolds which match to un-aligned *F. verticillioides* scaffolds; FXU LS, scaffolds which are absent in *F. verticillioides* but which are shared by *F. xylarioides*- and *F. udum* (FXU) or scaffolds which are unique to each *F. xylarioides* strain and are lineage-specific (LS). A High level functional diversity across the 11 syntenic chromosomes and scaffold groups based on the number of hits to each Level 1 SEED category in SUPER-FOCUS (Silva et al 2017); B Detailed functional diversity across chromosome 11 and the FXU and LS scaffold groups for *F. xylaroides* based on the number of hits to each Level 3 SEED category in SUPER-FOCUS. Abbreviations: PPCA, Predictions based on Plant Prokaryote Comparative Analysis; CVPP, Cofactors, Vitamins, Prosthetic Groups Pigments; PPTE, Phages, Prophages Transposable Elements; FALI, Fatty Acids, Lipds and Isoprenoids.

Table S1. Genome statistics for the *F. xylarioides* strains sequenced in this study, compared with sister species. Abbreviations for *Fusarium* sister species: *Fol, F. oxysporum f. sp. lycopersici; Fv, F. verticillioides*.

Name	*Coffea674*	*Coffea659*	Robusta277	Robusta254	Arabica563	Arabica908	Robusta925	Robusta394	*F. udum*	*Fol*	*Fv*
Strain number	IMI392674	IMI127659i	IMI392277	IMI392254	IMI389563	IMI375908i	IMI379925	FRC L-0394	F02845	4287	FGSC 7600
Reference	This study	This study	This study	This study	This study	This study	Olal et al. 2019	Wingfield et al. 2019	Srivastava et al. 2018	Ma et al. 2010	Ma et al. 2010
Date isolated	1951	1955	2003	1997	2002	1997	1998	2000	2010		
Origin	Cote d'Ivoire	CAR	Tanzania	Uganda	Ethiopia	Ethiopia	Uganda	Uganda	India		
Host	*Coffea*	*C. excelsa*	*C. c.* robusta	*C. c.* robusta	*C. arabica*	*C. arabica*	*C. c.* robusta	*C. canephora*	Pigeonpea	Tomato	Maize
Size (genome assembly) Mb	57.2	59.4	61.3	60.3	63.3	62.6	55.1	55.2	56.4	61.4	42.5
Genome size (Mb, >500bp)	54.4	55.1	56.5	56.3	58.0	57.8	55.1	55.2	56.4	61.4	42.5
Total repeats (genome assembly) Mb	13.3	14.9	15.9	15.3	18.6	17.8			12.6	10.8	1.0
Total repeats %	23	25	26	25	30	29			22	18	2
Interspersed repeats Mb	5.8	4.9	5.8	4.3	5.0	5.4			18.9	6.4	0.5
Interspersed repeats %	10	8	9	7	8	9			33	10.5	1
Interspersed repeats (raw reads) Mb	3.9	3.7	3.9	3.8	4.4	3.9				2.4	

Total repeats includes unclassified repeats. Interspersed repeats include retroelements, DNA transposons, simple and low complexity repeats

Table S2. Genome statistics for our strains mapped to the chromosomal assembly of *F. verticillioides* using reference-guided scaffolding. Abbreviations: FV, the contigs in each genome mapped to the syntenic chromosomes and unaligned scaffolds of *F. verticillioides*; FXU, scaffolds which are absent from *F. verticillioides* but which are present in *F. udum* and the historic *Coffea659* strain (*F. xylarioides* and *-udum* specific); FXS, scaffolds which are absent from *F. verticillioides* and *F. udum* and are shared with *Coffea659* (*F. xylarioides*-specific); LS, scaffolds which are not shared with *Coffea659* and are unique to each *F. xylarioides* strain (lineage-specific).

Strain	IMI392674	IMI127659i	IMI392277	IMI392254	IMI389563	IMI375908i
Name	Coffea674	Coffea659	robusta277	robusta254	arabica563	arabica908
Genome Size Mb	57.2	59.6	61.3	60.3	63.4	62.6
Total repeats incl unclassified Mb	13.3	14.9	15.9	15.3	18.6	17.8
Total repeats incl unclassified %	23	25	26	25	30	29
TEs Mb	5.8	4.9	5.8	4.3	5.0	5.4
TEs %	10	8	9	7	8	9
FV scaffolds Mb	51	49	51.6	51.3	52.4	52.3
FXU scaffolds Mb	4.5	7.9	7.5	7.2	8.9	7.7
FXS scaffolds Mb	0.04	-	0.06	0.04	0.04	0.5
LS scaffolds Mb	1.9	2.2	2.3	1.8	2.1	2.1
FV scaffolds %	7	5	5	5	6	6
FXU scaffolds %	8	13	12	12	14	12
FXS scaffolds %	0.1	-	0.1	0.1	0.1	0.8
LS scaffolds %	3	4	4	3	3	3
FV TEs Mb	3.6	2.4	2.8	2.4	2.9	3.2
FXU TEs Mb	1.2	1.9	1.8	1.4	1.6	1.7
LS TEs Mb	1.0	0.6	0.6	0.4	0.5	0.4
FV TEs %	7	5	5	5	6	6
FXU TEs (%)	28	24	23	20.1	18	22
LS TEs (%)	52	26	28	24	23	20

Table S3. Published genomes used for comparison

Genome	Accession number
F. udum	GCA_002194535.1
F. oxysporum f.sp. lycopersici	GCA_000149955.2
F. oxysporum f.sp. cubense	GCA_005930515.1
F. verticillioides	GCA_003316975.2
F. fujikuroi	GCA_900079805.1
F. mangiferae	GCA_900044065.1
F. solani	GCA_002215905.1
Verticillium dahliae	GCA_000150675.2
V. albo-atrum	GCA_002851705.1
F. graminearum	GCA_000240135.3
F. proliferatum	GCF_900067095.1
F. xylarioides IMI379925	GCA_004329255.1
F. xylarioides FRC L-0394	GCA_013183765.1

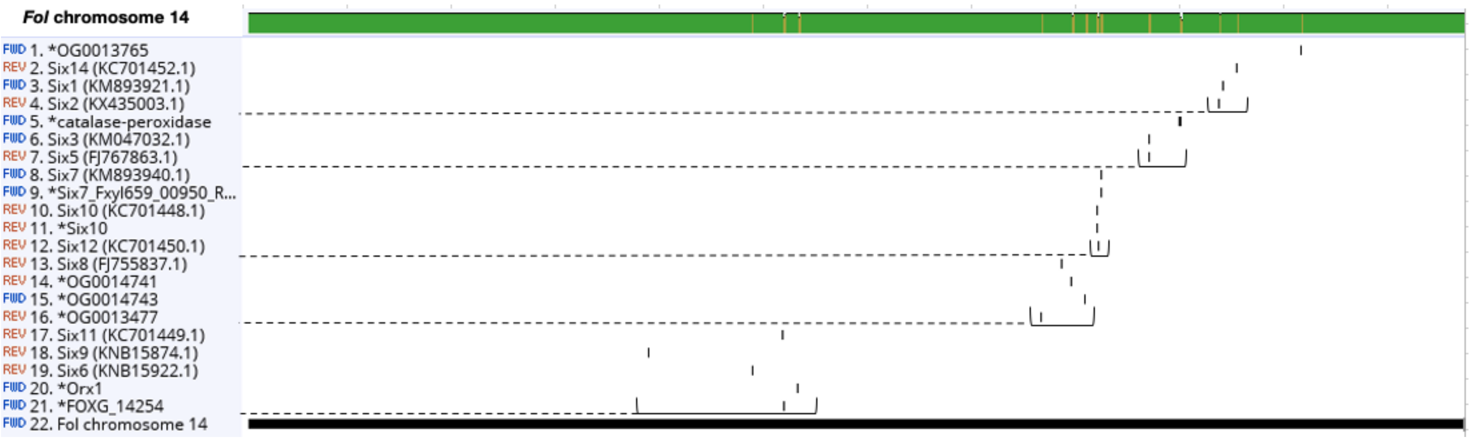

Fig. S6. Fol chromosome 14 aligned with all known SIX effectors and the nine effectors described in this study which match regions of chromosome 14 (prefixed with a *). Five SIX chromosomal mini-clusters (described by [**?**]) are marked on the genome plot. Eight effectors all reside close to four of these clusters: *FOXG_14254* and *Orx1* are 1.8 kb and 24 kb from *six11* (which is clustered with *six6* and five genes (including *orx1* in *Fol*) and a transcription factor); OG0013477, OG0014741 and OG0014743 are 30 kb, 12.5 kb and 33 kb respectively from *six8* (which resides in a solo block with class two transposons and a gene encoding an unknown protein); *six7* (from 659_00950) shares a locus with *Fol six7* and its cluster with *six10* and *six12*. The orthogroups OG0014741 and OG0014743 are both also <40 kb from this *six10*, *six12*, *six7* minicluster. Finally, catalase-peroxidase is 45 kb from the *six3*, *six5* minicluster. OG0013765 is 95 kb from the *six1*, *six2*, *six14* minicluster. Sequences aligned with MAFFT and drawn in Geneious 9.1.

Table S4. Predefined effector protein genes analysed in this study. Genes marked with an * show predicted roles and locations only

Name	Role	Query Accession	Query species	Length	Reference
FOXG_02706.2	Glucosyltransferase*	KNA98333.1	*Fol*	1493	[1]
FOXG_10732.2	Cytoskeletal*	KNB10567.1	*Fol*	449	[1]
FOXG_04660.2	Chloroplast/ vacuole*	KNB01401.1	*Fol*	797	[1]
Nep1	Microbial elicitors of plant necrosis	AF036580.1	*Foe**	2617	[2]
Fmk1	MAP kinase	KC257048.1	F. oxysporum	603	[3]
Fow1	Mitochondrial carrier protein	KC134256.1	F. oxysporum	725	[4]
Pda1	Pisatin demethylase	KR855811.1	F. oxysporum	455	[5]
PelA	Pectate lyase	MK918256.1	*Fol*	539	[6]
PelD	Pectate lyase	KC294608.1	F. proliferatum	552	[6]
Pep1	Pea pathogenicity protein	EU436568.1	Fusarium sp.	216	[7]
Rho1	Rho GTP-ase activating protein	KC017411.1	F. oxysporum	665	[8]
Sge1	SIX (secreted in xylem) gene expression 1	LC369105.1	*For**	565	[9]
Snf1	Protein kinase sucrose non-fermenting	KU048959.1	F. commune	625	[10]
FOXG_14254	Conserved secreted protein	KNB15932.1	*Fol*	1592	[11]
Orx1	In-planta secreted oxidoreductase enzyme	KNB15937.1	*Fol*	1860	[11]
Catalase-peroxidase	Secreted enzyme	KNB19974.1	*Fol*	2385	[11]
SIX10	Secreted in xylem 10	KNB20462.1	*Fol*	736	[11]

*Abbreviations for *F.oxysporum formae speciales* sister species: *Fol, F. oxysporum f. sp. lycopersici; Foe, F. oxysporum f. sp. erythroxyli; For, F. oxysporum f. sp. ricini*

Protein	Robusta		Coffea		Arabica		Transposon (bp from promoter)	Closest species match (BLAST)	F. oxysporum percent identity (%)
	Fx254	Fx277	Fx659	Fx674	Fx563	Fx908			
fow1								*F. fujikuroi*	
**pelD**	^	^	^	^	^	^		*F. verticillioides*	
fmk1								*F. oxysporum f. sp. lycopersici*	100
**sge1**								*F. acutatum*	
snf1								*F. verticillioides*	
**pep1**	"	"	"	"	"	"	216	*F. oxysporum f. sp. phaseoli*	94
chlo_vacu	^	^	^	^	^	^		*F. oxysporum*	89
rho1.1								*F. oxysporum f. sp. lycopersici*	100
rho1.2								*F. pseudograminearum*	
pelA	^	^	^	^	^	^		*F. oxysporum f. sp. lycopersici*	98
FOXG_14254	^	^	^	^	^	^		*F. anthophilum*	
**orx1**	^	^	^	^	^	^	235	*F. acutatum*	
**catalase-peroxidase**	^	^	^	^	^	^	916	*F. fujikuroi*	
nep1								*F. fujikuroi*	
glucosyltransferase								*F. fujikuroi*	
**pda1**								*F. oxysporum f. sp. pisi*	89
**six10**			"	"	"	"	1068	*F. oxysporum*	65
six7			^"		^"	^"		*F. oxysporum f. sp. lini*	90
**cytoskeletal**								*F. oxysporum f. sp. pisi*	87
OG13889a	^	^	^	^	^	^		*F. oxysporum*	85
OG13871a	^	^	^	^	^	^		*F. oxysporum*	90
OG13861	*	*	*	*	*	*		*F. poae*	
OG13877a	^	^	^	^	^	^		*F. langsethiae*	
OG14828a	*^	*^	*^	*^	*	*		*F. oxysporum*	77
**OG13477**	^~"	^~"	^~"	^~"	^~"	^~"	965	*F. oxysporum f. sp. pisi*	94
OG15372a	*^	*^	*^	*^	*^	*^		*F. oxysporum*	81
OG13645								*F. proliferatum*	
OG13792								*F. oxysporum*	40
**OG13763**	^	^	^	^	^	^		*Neonectria ditissima*	
**OG13738**	^~	^~		^~	^~	^~		*F. mangiferae*	
OG14864								*F agapanthi*	
**OG14398/Six7**	*^~"	*^~"		*^~"	*^~"	*^~"	369	*F. oxysporum*	63
OG14238	~"	~"		~"	~"	~"		*F. oxysporum f. sp. vasinfectum*	92
OG14165	*^	*^	*^	*^				*F. oxysporum f. sp. radici-cucunerinum*	74
OG15453	^	^	^	^				*F. beomiforme*	
OG14836a	^	^		^				*F. oxysporum f. sp. radici-cucunerinum*	77
OG16234	^	^	^						
OG13787			^	^	^	^		*F. acutatum*	
OG16323								*F. napiforme*	
OG14797	*^	*^			*^	*^		*F. anthophilum*	
OG14367a								*F. agapanthi*	
OG13912a	^	^	^		^	^		*F. nygamai*	
OG08649a								*F. oxysporum*	94
OG14891								*F. oxysporum*	82
OG16261								*F. oxysporum f. sp. cubense*	78
OG16232								*Trichoderma harzianum*	
**OG16241**								*F. oxysporum*	84
**OG16247**	~"	~"		~"				*F. oxysporum*	93
OG7097a	^	^	^	^				*F. fujikuroi*	
**OG14811**	^	^	^	^				*F. oxysporum f. sp. raphani*	83
**OG15465**	~	~	~	~				*F. nygamai*	
**OG14741**	*"	*"	*"	*"			246	*F. oxysporum f. sp. raphani*	99
**OG14743**	"	"	"	"			392	*F. oxysporum f. sp. raphani*	99
OG6324a	~	~	~	~				*F. oxysporum*	92
OG11333a	^	^	^	^	^	^		*F. fujiuroi*	
OG14180					*^"	*^"		*F. oxysporum*	90
**OG14392**	*~	*~	*~	*~	*^~	*^~	0	*F. oxysporum f. sp. pisi*	48
**OG13478**	"	"	"	"			451	*F. oxysporum f. sp. raphani*	98
**OG9441**							244	*F. oxysporum f. sp. raphani*	99
**OG973**							126	*F. oxysporum f. sp. vasinfectum*	99
**OG13765**	"	"	"	"			0	*F. oxysporum f. sp. cepae*	98
**OG15458**	"	"	"	"			746	*F. oxysporum*	99
**OG409**				^	^	^		*F. verticillioides*	
**OG14179**					^"	^"	1291	*F. oxysporum f. sp. radici-lycopersici*	97

Fig. S7. Putative effectors' characteristics and presence or absence across *F. xylarioides* strain and *F. udum*. The four effector classes are shown in: yellow for predefined effectors; purple for small and cysteine-rich secreted effectors; blue for carbohydrate-active enzymes; and red for transposon-adjacent effectors. The symbols highlight: the presence of transposons is represented by names in bold with its distance from the genes promoter described if less than 1500bp (if not, the transposon is over 1500bp away); genes under positive selection by an asterisk; genes in an AT-rich region by a tilde; genes with evidence of horizontal transfer from *F. oxysporum* are a darker shade; genes which are absent from more closely-related *Fusarium* species (namely *F. graminearum*, the Asian clade GFC species and *F. verticillioides* - *F. solani* and *F. udum* were excluded here because *F. solani* also infects coffee ([**?**] and thus could be a source of pathogenicity and *F. udum* is also a vascular wilt-inducer) and *F. oxysporum* is the closest match with a percent identity (%) >=90 are represented by a quotation mark; and closest species is shown for each protein with its percent identity (%), where a BLASTp hit was returned.

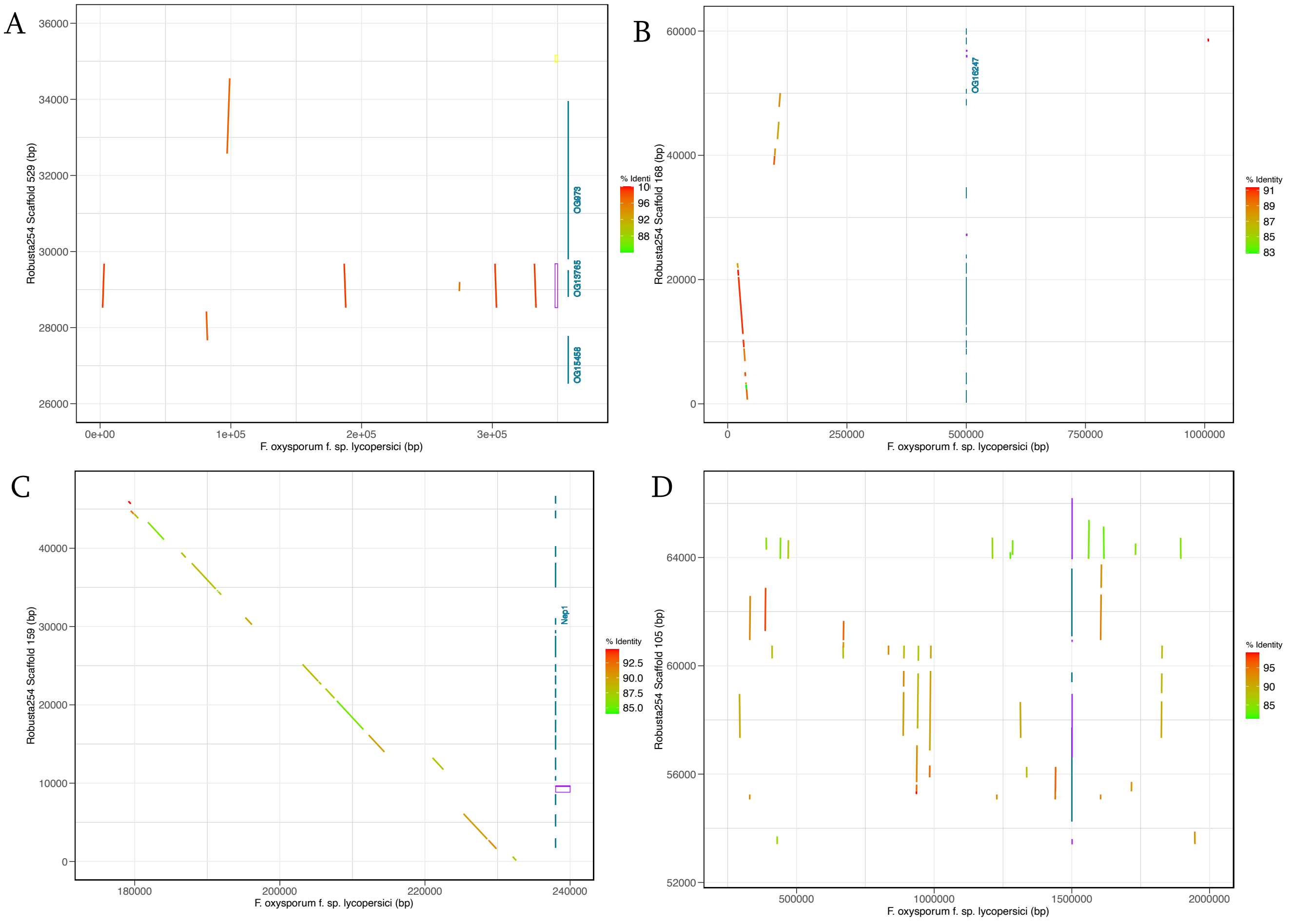

Fig. S8. Representative alignments of *F. xylarioides* robusta254 scaffolds which contain the putative 15 effectors under horizontal gene transfer against the genome of *F. oxysporum f. sp. lycopersici* (*Fol*). Line colour indicates the alignment percentage id match, x and y axes have been adjusted to display regions which match. Scaffolds were aligned using nucmer in the MUMmer3 package, with outputs processed in RStudio. An annotated blue line indicates a gene, an annotated and labelled blue line indicates a putative effector gene, yellow boxes indicate *mimps* and purple boxes indicate class 1 and class 2 transposable elements. A Scaffold 529 (35kb long) with three putative HGT effectors: OG15458, OG13785 and OG973; a *Hop3* DNA transposon and a *mimp*; B Scaffold 168 (79 kb) with 20 genes including one putative HGT effector OG16247, a *hAT* DNA transposon and two *Copia* retrotransposons; C Scaffold 159 (80 kb) with 18 genes including the putative HGT effector Nep1 and two *TcMar-Tc1* DNA transposons; D Scaffold 105 (96 kb) with three genes, four DNA transposons (*PiggyBac*, *TcMar-Tc1*, *MGR583-like*, *Fot6*.

Table S5. Enriched CAZyme gene familes across Fusarium species (*F. xylarioides*, *F. udum*, *Fol*, *F. verticillioides*, *F. fujikuroi*, *F. graminearum*) genomes, compared with three different ascomycete fungi (*Trichoderma reesei*, *Aspergillus nigris* and *Magnaporthe grisea*)

Species	AA*	CBM*	CE*	GH*	GT*	PL*
Fol	165	330	70	746	396	35
F. xylarioides (Coffea 674)	111	232	62	488	248	28
F. udum	119	228	63	503	257	30
F. verticillioides	130	265	66	597	336	28
F. fujikuroi	105	220	61	476	274	29
F. graminearum	92	191	52	394	241	27
Trichoderma reesei	57	127	25	304	196	8
Aspergillus nigris	94	151	55	434	321	10
Magnaporthe grisea	118	207	60	378	258	9

*Abbreviations: AA, Auxilliary Activities; CBM, Carbohydrate-Binding Modules; CE, Carbohydrate Esterase; GH, Glycoside Hydrolases; GT, Glycosyltransferases; PL, Pectate Lyases

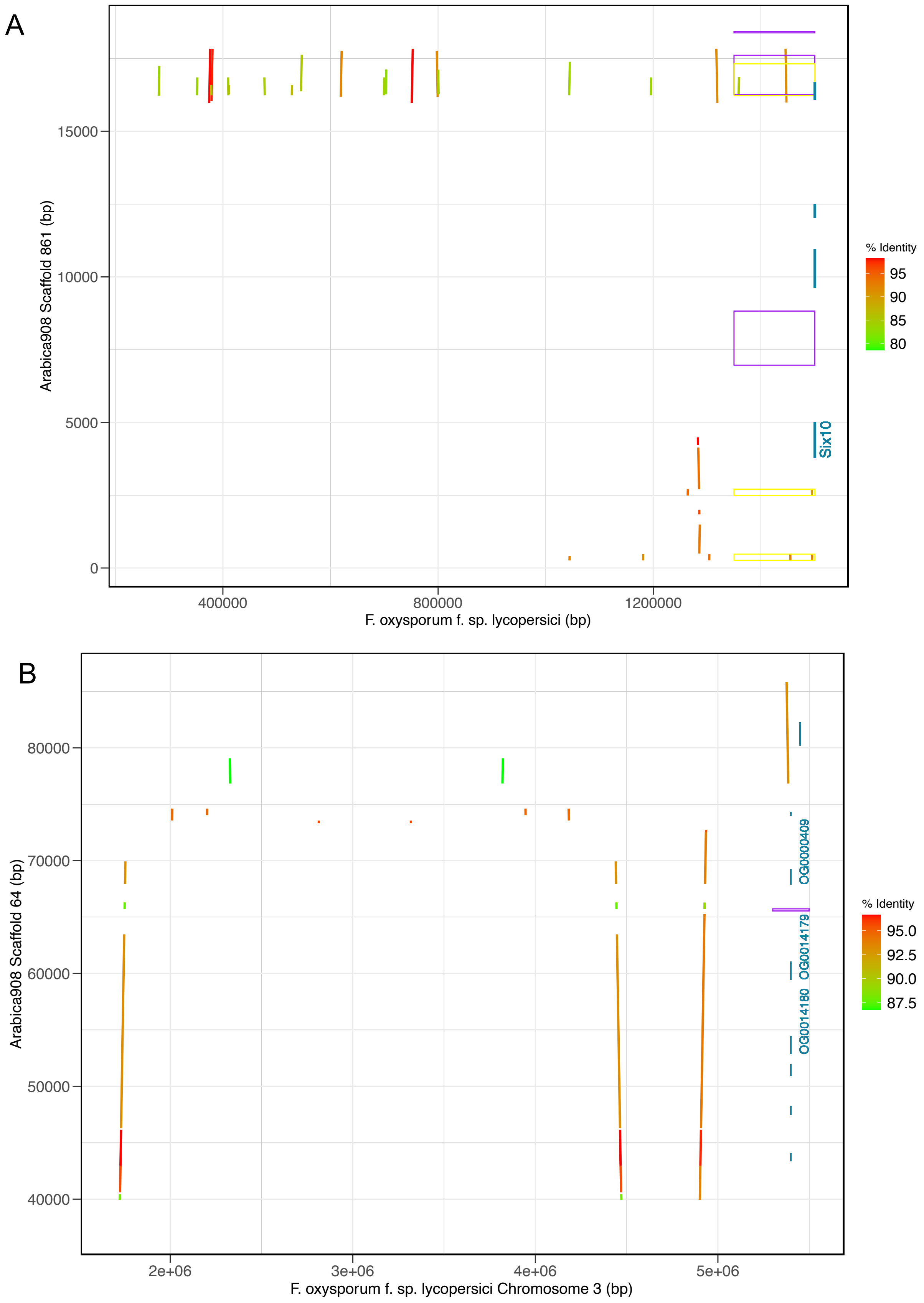

Fig. S9. Representative alignments of *F. xylarioides* arabica908 scaffolds which contain four of the effectors, unique to arabica and one, both or neither of the *Coffea* strains, under horizontal gene transfer, plotted against the genome of *F. oxysporum f. sp. lycopersici* (*Fol*). Line colour indicates the alignment percentage id match, x and y axes have been adjusted to display regions which match. Scaffolds were aligned using nucmer in the MUMmer3 package, with outputs processed in RStudio. An annotated blue line indicates a gene, an annotated and labelled blue line indicates a putative effector gene, yellow boxes indicate *mimps* and purple boxes indicate class 1 and class 2 transposable elements. **A** Scaffold 861 (20kb long) with the Six10 putative HGT effector, two *TcMar-Tc1* class II DNA transposons, one *Copia* retrotransposon and four *mimps*. **B** Scaffold 64 (93 kb long) with three putative HGT effectors: OG14180, OG14179, OG409 and a *Yaret2* class II DNA transposon.

CAZyme	*F. graminearum*	*F. mangiferae*	*F. proliferatum*	*F. solani*	*F. verticillioides*	*F. udum*	*Robusta254*	*Robusta277*	*Coffea659*	*Coffea674*	*Arabica563*	*Arabica908*	*Fol*	*Foc*	*V. albo-atrum*	*V. dahliae*	CAZy Genbank accession
						Vascular wilt-inducers											
CBM18					OG0013846		OG0013846	OG0013846	OG0013846	OG0013846	OG0013846	OG0013846					
CBM38				OG0008649		OG0008649	OG0008649<br>OG0008649<br>OG0018547	OG0008649<br>OG0008649<br>OG0018547	OG0008649<br>OG0018547	OG0008649<br>OG0018547	OG0008649<br>OG0018547	OG0008649<br>OG0018547	OG0008649<br>OG0008649<br>OG0008649	OG0008649	OG0008649	OG0008649	*F. fujikuroi*
CBM50					OG0013846		OG0013477	OG0013477	OG0013477	OG0013477	OG0013477<br>OG0013846	OG0013477<br>OG0013846	OG0013477				*Fol*<br>*Cordyceps militaris*
CBM66						OG0012516	OG0012516	OG0012516	OG0012516	OG0012516	OG0012516	OG0012516	OG0012516<br>OG0012516<br>OG0012516	OG0012516			*Bacillus*
GH35		OG0012085				OG0012085	OG0012085	OG0012085	OG0012085	OG0012085	OG0012085<br>OG0018531	OG0012085<br>OG0018531	OG0012085	OG0012085<br>OG0012085			
GH16							OG0013799	OG0013799	OG0013799		OG0013799	OG0013799	OG0013799	OG0013799			
GH28							OG0014847	OG0014847	OG0014847		OG0014847	OG0014847					*F. oxysporum*
GH29						OG0013912	OG0013912	OG0013912	OG0013912	OG0013912	OG0013912	OG0013912					
GH31							OG0016261<br>OG0018569	OG0016261<br>OG0018569	OG0016261<br>OG0018569	OG0018569							*Fusarium*
GH32				OG0008649<br>OG0012851		OG0008649<br>OG0012516<br>*	*<br>*<br>OG0012516<br>OG0012851	*<br>*<br>OG0012516<br>OG0012851	OG0008649<br>OG0012516<br>OG0012851	OG0008649<br>OG0012516<br>*	OG0008649<br>OG0012516<br>OG0012851	OG0008649<br>OG0012516<br>OG0012851	OG0008649<br>OG0008649<br>OG0008649<br>OG0012516<br>OG0012516<br>OG0012516<br>OG0012851	OG0008649<br>OG0012516<br>OG0012851	OG0008649	OG0008649	
GH78						*	OG0013475	OG0013475<br>*	OG0013475<br>*	*	*<br>OG0014891	*<br>OG0014891	OG0013475	OG0013475			
GT1			OG0013486			*	OG0013486<br>OG0014741<br>OG0014743	OG0013486<br>OG0014741<br>OG0014743	OG0013486<br>OG0014741<br>OG0014743	OG0013486<br>OG0014741<br>OG0014743	OG0013486	OG0013486	OG0014741<br>OG0014743				*Fremyella diplosiphon*<br>*Hormonema carpetanun*
GH3							OG0015465	OG0015465	OG0015465	OG0015465							
GT2							OG0014861	OG0014861	OG0014861	OG0014861		OG0014861			OG0014861		
GH43_11							OG0016241	OG0016241	OG0016241								
GT21							OG0016232	OG0016232	OG0016232								*Pyricularia oryzae*
GH43_24		OG0013753				OG0013753	OG0013753	OG0013753	*	OG0013753			OG0013753				*Plantactinospora*
CBM13		OG0013753				OG0013753	OG0013753	OG0013753	*	OG0013753			OG0013753				*Streptomyces*
CBM35		OG0013753				OG0013753	OG0013753	OG0013753	*	OG0013753			OG0013753				
GH5_16						*	OG0013130	OG0013130	*	OG0013130	*	*	OG0013130	OG0013130			
GT0							OG0014861	OG0014861	*	OG0014861		OG0014861					
GH88								OG0018497		OG0018497							*F. fujikuroi*
CE16		OG0015193					OG0015193	OG0015193		OG0015193							
CBM1		OG0015193					OG0015193	OG0015193		OG0015193							
GH134							OG0016247	OG0016247		*							*Pseudogymnoascus*
CE8							OG0018533	OG0018533	OG0018533	OG0018533							*F. fujikuroi*
CE5							OG0014836	*<br>*<br>OG0014836		*							*F. fujikuroi*
CBM42		OG0013419				OG0013419			OG0013419	OG0013419	OG0013419	OG0013419			OG0013419	OG0013419	
GH43_26		OG0013419				OG0013419			OG0013419	OG0013419	OG0013419	OG0013419			OG0013419	OG0013419	
GH67					OG0014678					OG0014678	OG0014678	OG0014678	OG0014678				
GT22							*	*	*	*	OG0014367	OG0014367					
CBM67								*	*	*	OG0014891	OG0014891					*Streptomyces*
GH18											OG0014180	OG0014180	OG0014180<br>OG0014180<br>OG0014180<br>OG0014180				*Trichoderma / F. fujikun*
PL1_4						OG0016212					OG0016212	OG0016212					*F. fujikuroi*

Fig. S10. Gene copy number for CAZyme-encoding orthologous groups shared across the vascular wilt-inducing *Fusarium* and *Verticillium*. Groups which also included one non-vascular wilt inducer were additionally included, and those which are also a putative effector are shaded the same colour as in figure 5. Where a species has a gene in the orthologous group which is not recognised as a CAZyme is represented with an asterisk.

Table S6. Accession numbers and source details for each *impala*, *miniature impala (mimp)* and newly described class II transposable elements

Accession	Transposon	Sequence
AF076624.1	F. o. repetitive element mimp1	
AF076625.1	F. o. repetitive element mimp2	
EU833100.1	F. o. f. sp. melonis MITE mimp3	complete sequence
EU833101.1	F. o. f. sp. lycopersici MITE mimp4	complete sequence
AF282722.1	F. o. f. sp. melonis transposon impala transposase gene	complete cds
AF363407.1	F. o. f. sp. melonis transposon impala M24-impE	partial sequence
AF363412.1	F. o. f. sp. lini transposon impala Ln3-1	partial sequence
AF363413.1	F. o. f. sp. lini transposon impala Ln88-23	partial sequence
AF363414.1	F. o. f. sp. cubense transposon impala Cu-12	partial sequence
AF363416.1	F. o. f. sp. phaseoli transposon impala Ph-5	partial sequence
AF363417.1	F. o. f. sp. phaseoli transposon impala Ph-9	partial sequence
AF363418.1	F. o. f. sp. albedinis transposon impala A-33	partial sequence
AF363419.1	F. o. f. sp. soil transposon impala S47-35	partial sequence
AF363420.1	F. o. f. sp. raphani transposon impala R-8	partial sequence
AF363425.1	F. o. f. sp. melonis transposon impala M24-impD	partial sequence
AF363426.1	F. o. f. sp. melonis transposon impala MK14	partial sequence
AF363427.1	F. o. f. sp. lini transposon impala Ln88-8	partial sequence
AF363428.1	F. o. f. sp. radicis-lycopersici transposon impala RL28delta22	partial sequence
AF363429.1	F. o. f. sp. melonis transposon impala MKdelta208	partial sequence
AF363430.1	F. o. f. sp. lycopersici transposon impala L15delta5	partial sequence
AF363432.1	F. o. f. sp. lycopersici transposon impala L15-15	partial sequence
AF363433.1	F. o. f. sp. radicis-lycopersici transposon impala RL28-17	partial sequence
AF363434.1	F. o. f. sp. lini transposon impala Ln86-10	partial sequence
AF363435.1	F. o. f. sp. ciceris transposon impala Ci-36	partial sequence
AF363436.1	F. o. f. sp. ciceris transposon impala Ci-16	partial sequence
AF363437.1	F. o. f. sp. melonis transposon impala MK28	partial sequence
AF363438.1	F. o. f. sp. lycopersici transposon impala L15-16	partial sequence
AJ608703.3	F. o. f. sp. lycopersici shh1 gene	
AJ608703.3	F. o. f. sp. lycopersici fot5 gene	
JX204302.1	F. o. f. sp. fragariae transposon $Impala_1$	complete sequence
Schmidt et al 2013	FoCrypton	
Schmidt et al 2013	FoHelitron	
Schmidt et al 2013	Fot6	
Schmidt et al 2013	Fot8	
Schmidt et al 2013	Hop3	
Schmidt et al 2013	Hop6	
Schmidt et al 2013	MGR583-like	
Schmidt et al 2013	Nht2-like	
Schmidt et al 2013	YahAT4	
Schmidt et al 2013	YahAT6	
Schmidt et al 2013	Yaret1	
Schmidt et al 2013	Yaret2	

Table S7. The median overlap distance to transposable elements and Large RIP Affected Areas (LRAR) for our putative effectors and the same number of random genes (randomisation trials, repeated 1000 times, * = p < 0.05) for each *F. xylarioides* strain.

	Random genes		Putative effectors		P-value	
	Transposons (kb)	LRAR (kb)	Transposons (kb)	LRAR (kb)	Transposons (kb)	LRAR (kb)
Robusta254	6.43	22.39	4.00	15.16	p > 0.05	*
Robusta277	5.79	22.45	2.48	16.69	*	p > 0.05
Coffea659	6.17	24.94	3.00	14.07	p >0.05	p > 0.05
Coffea674	7.41	26.99	2.97	22.50	*	p > 0.05
Arabica563	5.26	18.54	2.16	7.51	*	*
Arabica908	4.59	18.14	3.66	6.46	p > 0.05	*

Table S8. Investigating the support for horizontal acquisition for each putative effector gene in *F. xylarioides (Fx)* with *F. oxysporum (Fo)* as the source of pathogenicity. Following the decision tree in figure (*), this table reports the outcome for each stage of the decision tree. The stages are: is the effector present in other FFC species (Y / N); if Y, does Fx nest with the FFC gene copies (Y / N); if N, does Fx nest with Fo thus disrupting the Fo phylogeny (Y / N); branch support values (BSV) for Fx with Fo (n); if N, does Fx nest with Fo by distance i.e. less distance from Fx to Fo than greatest distance from Fo to Fo; pairwise id % for whole branch with support value for Fx nested with Fo; the HGT class that we assign, classes 2 and 3 display evidence of HGT. FFC = *F. fujikuroi* complex that *F. xylarioides* belongs to.

Effector	Effector present in FFC	Fx nested with FFC	BSV	Fx nested in Fo by phylogeny	BSV	Fx nested in Fo by distance	Pairwise id %	Class
fow1	Y	Y		N				1
pelD	Y	Y		N				1
fmk1	Y	Y		N				1
sge1	Y	Y		N				1
snf1	Y	Y		N				1
pep1	N	N		Y	91		94	3a
chlo_vacu	Y	Y		N				1
rho1.1	Y	Y		N				1
rho1.2	Y	Y		N				1
pelA	Y	Y		N				1
FOXG_14254	Y	Y		N				1
orx1	Y	Y		N				1
catalase-peroxidase	Y	Y		N				1
nep1	Y	N		Y	99		92	2
gluco	Y	Y		N				1
pda1	Y	Y		N				1
six10	N	N		N	100	Y	84	3b
six7	N	N		N			91	4
cytoskeletal	Y*	Y						1
OG13899	Y	Y*	100	N				1
OG13871	Y	Y		N				1
OG13861	Y	Y		N				1
OG13877	Y	Y*		N				1
OG14828	Y	Y		N				1
OG13477	N	N		Y	100		84.5	3a
OG15372	Y	Y		N				1
OG13645	N	no blast match		N				5
OG13792	Y	Y		N				1
OG13763	N	no blast match		N				5
OG13738	Y	Y		N				1
OG14864	Y	Y		N				1
OG14398	N	N					84.4	4
OG14238	N	N		N			88.9	4
OG14165	Y	Y*		N				1
OG15453	N	no blast match		N				5
OG14836	Y	Y*		Y	100		65.8	1

OG16234	N	no blast match		N				5
OG13787	Y	Y*	80	N				1
OG16323	N	no blast match		N				5
OG14797	Y	Y*	99	N				1
OG14367	N	no blast match		N				5
OG13912	Y	Y						1
OG08649	Y	Y*		Y	100		88.7	1
OG14891	Y	Y		N				1
OG16261	N	Weak Fo blast hit		N				5
OG16232	Y	Y		N				1
OG16241	Y	N		Y	100		89.1	2
OG16247	N	N		N		Y	80.4	3b
OG7097	Y	Y		N				1
OG14811	Y	Y						1
OG15465	Y	N		Y	100		88	2
OG14741	N	N		Y	100	Y	90.1	3a
OG14743	N	N		N		Y	97.9	3b
OG16212	Y	Y						1
OG006324	Y	Y		N				1
OG11333	Y	Y		N				1
OG14180	N	N		N		Y	95.6	3b
OG14392	Y	Y*	46	N				1
OG13478	N	N		N	100	Y	98.8	3b
OG09441	Y	Y/N		N				2
OG00973	Y	N		Y	100		83.6	2
OG13765	N	N		Y	100		86.5	3b
OG15458	N	N		N				4
OG00409	Y	Y						1
OG14179	N	N		Y	99		82	3a

Table S9. BioProject accession numbers

Name	Species	Strain	BioProject	BioSample	Locus tag	Reads	Bases	Genome size	Coverage	Run accessions
Coffea674	Fusarium xylarioides	IMI 392674	PRJNA659227	SAMN15901422	H9Q69	2,649,496	763,216,608	57,075,484	13.4	SRR12534416
Coffea659	Fusarium xylarioides	IMI 127659i	PRJNA659227	SAMN15901423	H9Q72	2,207,606	634,942,231	59,379,521	10.7	SRR12534415
Robusta277	Fusarium xylarioides	IMI 392277	PRJNA659227	SAMN15901435	H9Q70	3,330,016	959,882,147	61,161,285	15.7	SRR12534414
Robusta254	Fusarium xylarioides	IMI 392254	PRJNA659227	SAMN15901436	H9Q73	2,614,366	753,518,666	60,101,568	12.5	SRR12534413
Arabica563	Fusarium xylarioides	IMI 389563	PRJNA659227	SAMN15901606	H9Q71	2,633,060	759,347,660	63,111,562	12	SRR12534412
Arabica908	Fusarium xylarioides	IMI 375908i	PRJNA659227	SAMN15901615	H9Q74	2,374,418	684,973,154	62,330,925	11	SRR12534411

REFERENCES

1. S. J. Klosterman, K. V. Subbarao, S. Kang, P. Veronese, S. E. Gold, B. P. Thomma, Z. Chen, B. Henrissat, Y. H. Lee, J. Park, M. D. Garcia-Pedrajas, D. J. Barbara, A. Anchieta, R. de Jonge, P. Santhanam, K. Maruthachalam, Z. Atallah, S. G. Amyotte, Z. Paz, P. Inderbitzin, R. J. Hayes, D. I. Heiman, S. Young, Q. Zeng, R. Engels, J. Galagan, C. A. Cuomo, K. F. Dobinson, and L. J. Ma, "Comparative genomics yields insights into niche adaptation of plant vascular wilt pathogens," PLoS Pathog. **7** (2011).
2. C. L. Pemberton and G. P. Salmond, "The Nep1-like proteins - A growing family of microbial elicitors of plant necrosis," Mol. Plant Pathol. **5**, 353–359 (2004).
3. A. Di Pietro, F. I. García-Maceira, E. Méglecz, and M. I. G. Roncero, "A MAP kinase of the vascular wilt fungus Fusarium oxysporum is essential for root penetration and pathogenesis," Mol. Microbiol. **39**, 1140–1152 (2004).
4. I. Inoue, F. Namiki, and T. Tsuge, "Plant Colonization by the Vascular Wilt Fungus Fusarium oxysporum Requires FOW1 , a Gene Encoding a Mitochondrial Protein," The Plant Cell **14**, 1869–1883 (2002).
5. C. Wasmann and H. VanEtten, "Transformation-mediated chromosome loss and disruption of a gene for pisatin demethylase decrease the virulence of Nectria haematococca on pea," Mol. Plant-Microbe Interactions (USA) (1996).
6. L. M. Rogers, Y.-K. Kim, W. Guo, L. Gonzá Lez-Candelas, D. Li, P. E. Kolattukudy, and C. A. Ryan, "Requirement for either a host-or pectin-induced pectate lyase for infection of Pisum sativum by Nectria hematococca," Natl. Acad Sci. **97** (2000).
7. Y. Han, X. Liu, U. Benny, H. Corby Kistler, and H. D. VanEtten, "Genes determining pathogenicity to pea are clustered on a supernumerary chromosome in the fungal plant pathogen Nectria haematococca," Plant J. **25**, 305–314 (2001).
8. A. L. Martínez-Rocha, M. I. G. Roncero, A. López-Ramirez, M. Mariné, J. Guarro, G. Martínez-Cadena, and A. Di Pietro, "Rho1 has distinct functions in morphogenesis, cell wall biosynthesis and virulence of Fusarium oxysporum," Cell. Microbiol. **10**, 1339–1351 (2008).
9. C. B. Michielse and M. Rep, "Pathogen profile update: Fusarium oxysporum," Mol. Plant Pathol. **10**, 311–324 (2009).
10. M. D. Ospina-Giraldo, E. Mullins, and S. Kang, "Loss of function of the Fusarium oxysporum SNF1 gene reduces virulence on cabbage and Arabidopsis," Curr. Genet. **44**, 49–57 (2003).
11. L. J. Ma, H. C. Van Der Does, K. A. Borkovich, J. J. Coleman, M. J. Daboussi, A. Di Pietro, M. Dufresne, M. Freitag, M. Grabherr, B. Henrissat, P. M. Houterman, S. Kang, W. B. Shim, C. Woloshuk, X. Xie, J. R. Xu, J. Antoniw, S. E. Baker, B. H. Bluhm, A. Breakspear, D. W. Brown, R. A. Butchko, S. Chapman, R. Coulson, P. M. Coutinho, E. G. Danchin, A. Diener, L. R. Gale, D. M. Gardiner, S. Goff, K. E. Hammond-Kosack, K. Hilburn, A. Hua-Van, W. Jonkers, K. Kazan, C. D. Kodira, M. Koehrsen, L. Kumar, Y. H. Lee, L. Li, J. M. Manners, D. Miranda-Saavedra, M. Mukherjee, G. Park, J. Park, S. Y. Park, R. H. Proctor, A. Regev, M. C. Ruiz-Roldan, D. Sain, S. Sakthikumar, S. Sykes, D. C. Schwartz, B. G. Turgeon, I. Wapinski, O. Yoder, S. Young, Q. Zeng, S. Zhou, J. Galagan, C. A. Cuomo, H. C. Kistler, and M. Rep, "Comparative genomics reveals mobile pathogenicity chromosomes in Fusarium," Nature **464**, 367–373 (2010).
